# Mathematical foundations of artificial intelligence in healthcare: a systematic review

**DOI:** 10.3389/frai.2026.1995929

**Published:** 2026-10-21

**Authors:** Yuliana Jiménez-Gaona, Vasudevan Lakshminarayanan

**Affiliations:** 1 Departamento de Química y Producción, Universidad Técnica Particular de Loja, Loja, Ecuador; 2 Theoretical and Experimental Epistemology Lab, School of Optometry and Vision Science, University of Waterloo, Waterloo, ON, Canada; 3 Department of Systems Design Engineering, Physics, and Electrical and Computer Engineering, University of Waterloo, Waterloo, ON, Canada

**Keywords:** artificial intelligence, ethic and development, explainable AI, healthcare, mathematical foundations

## Abstract

Artificial intelligence (AI) in healthcare relies on diverse mathematical frameworks, yet their contributions to model development, evaluation, interpretability, and clinical translation remain fragmented. This systematic review synthesized the classical and emerging mathematical foundations underpinning healthcare AI and examined major methodological and application trends. Following PRISMA 2020 guidelines, studies published between January 2016 and June 30, 2026, were identified through systematic searches of Scopus, Google Scholar, and PubMed/MEDLINE, with searches conducted using Harzing’s Publish or Perish v8 where applicable. Bibliometric analysis was performed using VOSviewer, while methodological quality was assessed using an adapted JBI-based framework and AMSTAR-2 for systematic reviews. Of 653 records identified, 147 studies met the eligibility criteria. Non-mutually exclusive mathematical-domain coding identified linear algebra in 100/147 studies (68.0%), calculus and optimization in 91/147 (61.9%), and probability and statistics in 79/147 (53.7%), highlighting their roles in data representation, learning, optimization, inference, uncertainty quantification, and validation. A separate mutually exclusive classification of dominant methodological themes identified explainable and trustworthy AI in 24/147 studies (16.3%), statistics and learning theory in 23/147 (15.6%), probability and Bayesian methods in 23/147 (15.6%), calculus and optimization in 19/147 (12.9%), and linear algebra in 15/147 (10.2%). Radiology and medical imaging were the leading healthcare application (24.3%), while classification and diagnosis were the most frequent clinical task (17.1%). Overall, healthcare AI is supported by an interconnected mathematical ecosystem. Emerging approaches, including graph neural networks, Transformers, and quantum machine learning, require stronger external validation, standardized benchmarking, and rigorous evaluation for reliable clinical translation.

## 1 Introduction

Artificial intelligence (AI) has become increasingly important in healthcare, enabling advances in medical imaging, disease diagnosis, clinical decision support, drug discovery, personalized medicine, and healthcare management (Stone, 2024; Oliveira and Teodoro, 2024; Kaur and Sharma, 2025; Michelucci, 2024). Developments in machine learning, deep learning, natural language processing, and computer vision have improved the analysis of large-scale and multimodal biomedical data, with some models achieving expert-level performance in specific clinical tasks (Pantsar, 2024; Ananthaswamy, 2024; Kottahachchi Kankanamge Don and Khalil, 2025; Pira and Ferrie, 2024). Consequently, AI plays an important role into diagnostic workflows, precision medicine, and digital health applications to support clinical decision-making (Gühring et al., 2022; Parekh and Jacobs, 2019; Barredo Arrieta et al., 2020).

Despite these advances, the translation of AI into routine clinical practice remains constrained by important methodological and clinical challenges. Many state-of-the-art models operate as “black boxes,” providing predictions without sufficient interpretability or transparency for high-stakes clinical decisions (Wei et al., 2023; Kim et al., 2025; Zhang, 2022). Their reliability may also be affected by data heterogeneity, class imbalance, measurement uncertainty, limited generalizability across populations, and algorithmic bias (Fronckova et al., 2018; Symeonidis and Zioupos, 2016; Rani, 2024; Goodfellow et al., 2016). Additional barriers include limited availability of annotated medical datasets, computational complexity, multimodal data integration, and increasing requirements for explainability, fairness, robustness, reproducibility, and patient safety (LeCun et al., 2015; Bishop and Bishop, 2024; Murphy, 2022; Bronstein et al., 2017). These limitations indicate that predictive performance alone is insufficient to ensure trustworthy and clinically deployable AI.

Addressing these challenges requires a deeper understanding of the mathematical principles governing AI development, optimization, validation, and interpretation. Mathematics provides the formal framework through which AI models represent information, learn from data, quantify uncertainty, and evaluate robustness and reproducibility (Hamilton, 2020; Hogan et al., 2021; Russell and Norvig, 2021) (Figure 1). For conceptual consistency, the mathematical foundations identified in this review were organized into eighth overarching domains.

**Figure 1:**
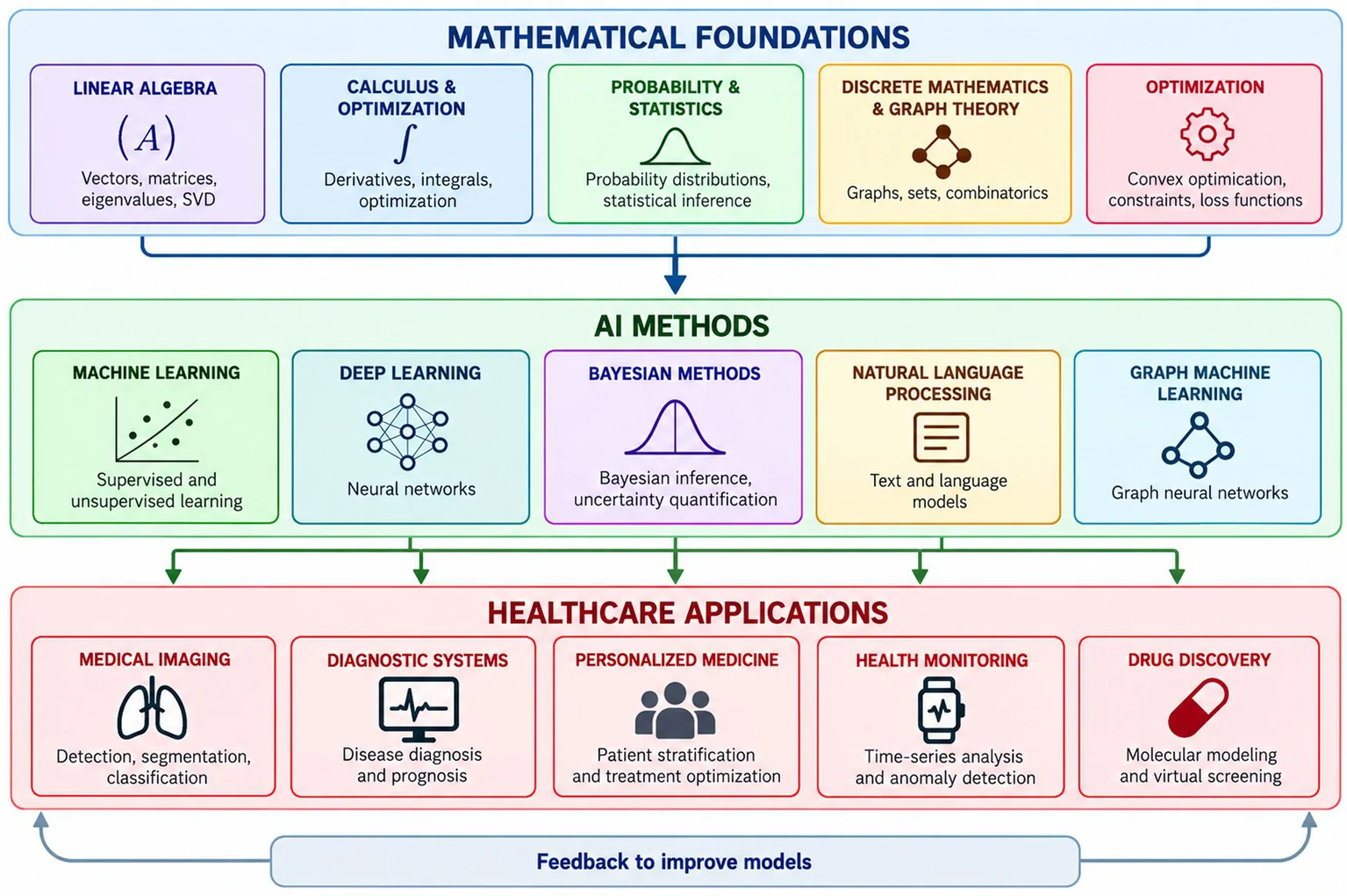
Conceptual framework linking mathematical foundations with artificial intelligence architectures, healthcare applications, explainability, uncertainty, robustness, and clinical reliability.

These domains were defined as an analytical taxonomy rather than as mutually exclusive categories, because contemporary AI architectures typically integrate multiple mathematical foundations within the same computational pipeline. For example, deep neural networks combine linear algebra for data representation, calculus and optimization for parameter learning, and probability and statistics for inference, uncertainty quantification, and validation. This taxonomy therefore provides a consistent framework for examining how mathematical principles support AI architectures, healthcare applications, and trustworthy AI properties throughout this review.

Linear algebra enables high-dimensional data representation and transformations; calculus and optimization govern model learning and parameter estimation; probability and statistics support inference and uncertainty quantification; graph theory represents complex biomedical relationships; and information theory characterizes information content and learning efficiency. Emerging areas, including topology, geometry, stochastic methods, and quantum computing, are further extending the analytical capabilities of AI systems (Harzing, 2007; Van Eck and Waltman, 2010; Aromataris et al., 2024; Shea et al., 2017). Collectively, these mathematical foundations influence not only computational performance but also model interpretability, reliability, and potential for safe clinical implementation.

Previous reviews have examined important components of healthcare AI, including deep learning, optimization, explainable AI, radiomics, graph neural networks, federated learning, and quantum machine learning (Boers, 2018; Mayo-Wilson et al., 2018; Stovold et al., 2014; Suh and Cheng, 2024). However, these studies generally focus on specific computational approaches, mathematical domains, or clinical applications. Consequently, the relationships among classical and emerging mathematical foundations, contemporary AI architectures, healthcare applications, and the requirements of trustworthy clinical AI remain insufficiently integrated.

### 1.1 Research gap, novelty, and objectives

Although the mathematical foundations of AI have represented in the reviewed literature, existing evidence remains fragmented across theoretical, computational, and clinical domains. Previous reviews have provided valuable but largely specialized perspectives. Gühring et al. (2022) examined mathematical principles underlying modern AI, including approximation, optimization, expressivity, and generalization, but did not focus specifically on healthcare applications or clinical translation. Parekh and Jacobs (2019) reviewed deep learning and radiomics in precision medicine, emphasizing diagnostic, prognostic, and imaging applications while providing comparatively limited integration of the broader mathematical foundations governing learning, uncertainty, and robustness. Barredo Arrieta et al. (2020) established an influential framework for explainable AI but primarily addressed interpretability and transparency rather than the broader mathematical mechanisms supporting uncertainty estimation, calibration, fairness, and model reliability. More recently, Wei et al. (2023) examined quantum machine learning for medical image analysis, but this emerging paradigm was considered independently from classical mathematical domains such as linear algebra, probability, optimization, graph theory, and information theory.

Therefore, a significant gap remains: the mathematical foundations of healthcare AI have not been comprehensively synthesized within a unified framework linking mathematical theory, AI architectures, healthcare applications, and the requirements of trustworthy and clinically translatable AI. In particular, the collective contributions of linear algebra, calculus and optimization, probability and statistics, information theory, graph theory, stochastic processes, fuzzy logic, topology, differential equations, random matrix theory, and quantum computing remain insufficiently characterized in relation to explainability, uncertainty quantification, robustness, transparency, and clinical reliability.

To address this gap, this study conducts a PRISMA-based (Page et al., 2021) systematic review complemented by bibliometric analysis. Unlike previous reviews centered on individual mathematical domains, computational techniques, or medical applications, the present study provides an integrated framework connecting mathematical foundations, AI methodologies and architectures, healthcare applications, trustworthy AI properties. This integrative perspective represents the principal novelty of the review and is intended to clarify how mathematical rigor contributes to the development, evaluation, and potential clinical translation of AI systems.

Accordingly, this review aims to: (i) identify and analyze the principal mathematical foundations supporting contemporary AI methods in healthcare; (ii) examine their contributions to model learning, performance, uncertainty estimation, explainability, robustness, and clinical decision-making; (iii) characterize their relationships with AI architectures and healthcare applications; and (iv) identify methodological gaps and emerging mathematical directions relevant to trustworthy and clinically translatable healthcare AI.

#### 1.1.1 Research questions

- RQ1. Which mathematical domains most strongly underpin contemporary AI applications in healthcare?
- RQ2. How are mathematical foundations associated with different AI architectures and healthcare applications?
- RQ3. How do classical and emerging mathematical frameworks contribute to explainability, uncertainty estimation, robustness, and clinical reliability?
- RQ4. What methodological gaps and emerging mathematical directions currently shape the development of trustworthy and clinically translatable healthcare AI?

## 2 Methodology

### 2.1 Study design

The systematic review was conducted in accordance with the PRISMA 2020 (Page et al., 2021; Yepes-Nuñez et al., 2021) guidelines and followed a predefined sequential workflow comprising: (1) Database sources and searching strategies, (2) Boolean search implementation, (3) Record deduplication, (4) Eligibility criteria, (5) Screening and study selection, (6) Methodological quality assessment, (7) Evidence synthesis, and (8) Bibliometric analysis. The review focused on identifying and synthesizing the mathematical foundations underpinning contemporary artificial intelligence methods and their applications in healthcare (see Figure 2).

**Figure 2:**
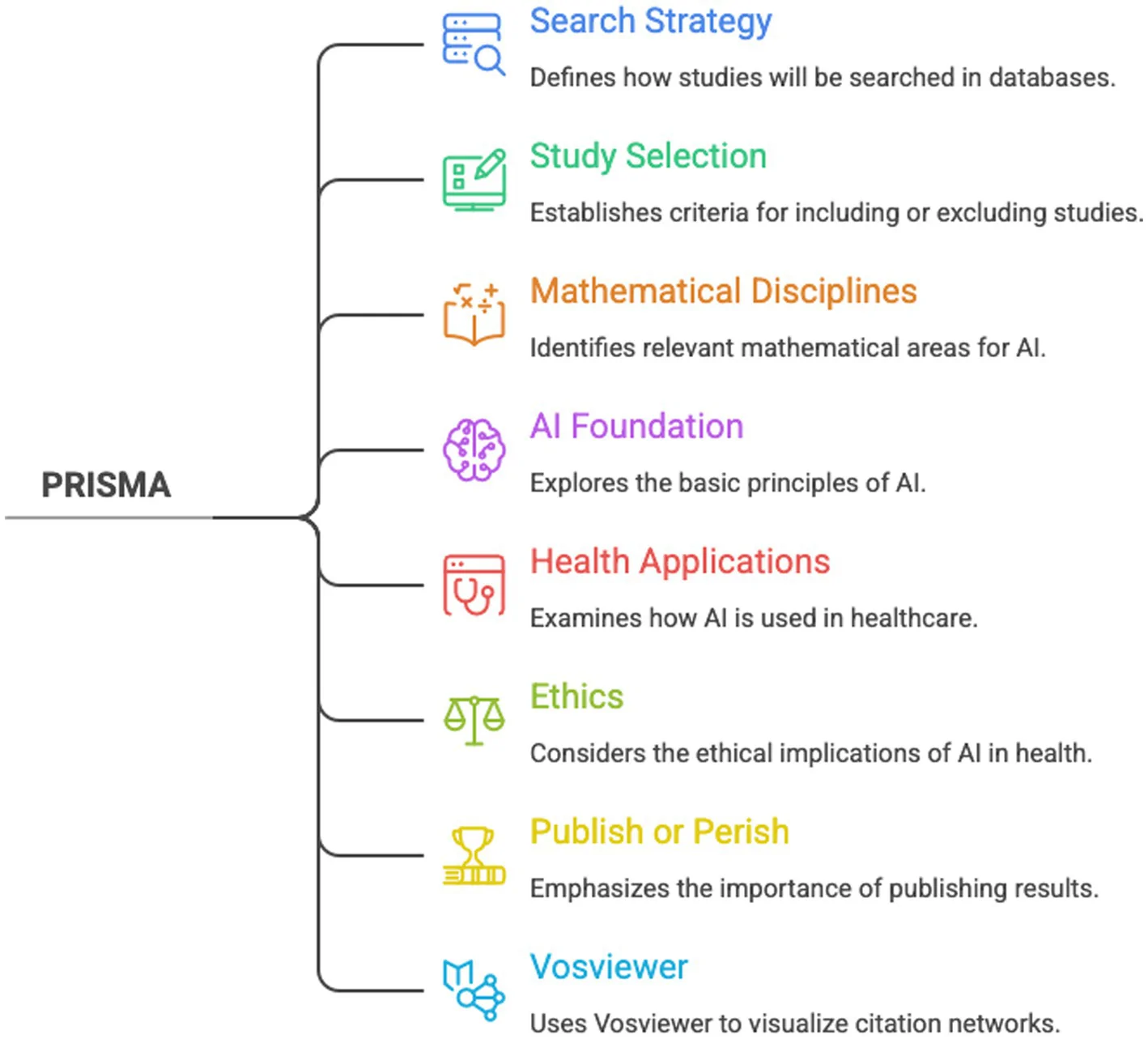
Methodology workflow to construct a systematic review. Design by Napkin https://app.napkin.ai.

### 2.2 Databases and search strategy

A systematic literature search was conducted using Scopus and Google Scholar, with Google Scholar, PubMed searches performed through Harzing’s Publish or Perish (PoP) v8. The search covered publications from January 1, 2016, to June 30, 2026. The search strategy comprised three predefined conceptual blocks: (1) artificial intelligence methods, (2) mathematical foundations, and (3) healthcare applications. Database-specific search expressions were adapted to the syntax and indexing characteristics of each source while preserving the same conceptual structure. The complete search strategies, search fields, filters and restrictions, search dates, and number of records retrieved from each source are reported in Table 1 and Appendix A. The final RIS file with 147 studies were unique at the DOI and/or title level.

**Table 1:** Information sources, search parameters, and records retrieved.

Information source and search platform	Search strategy/fields	Query	Records retrieved, *n*
Scopus (Elsevier)	TITLE-ABS-KEY; Boolean combinations of AI, mathematical-foundation, and healthcare terms	TITLE-ABS-KEY ((“artificial intelligence” OR “machine learning” OR “deep learning”) AND (mathematics foundations OR “linear algebra” OR calculus OR optimization OR probability OR statistics OR “graph theory” OR “information theory” OR “numerical analysis” OR “quantum computing”) AND (healthcare OR medicine OR biomedical OR radiology OR radiomics)) AND PUBYEAR >2015	258
Google Scholar (PoP v8)	Source-adapted combinations of AI, mathematical-foundation, and healthcare terms.	(“artificial intelligence” OR “machine learning” OR “deep learning”) AND (“mathematical foundations” OR “linear algebra” OR calculus OR optimization OR probability OR statistics OR “graph theory” OR “information theory” OR “numerical analysis” OR “quantum computing”) AND (healthcare OR medicine OR biomedical OR radiology OR radiomics)	342
PubMed/MEDLINE (PoP v8)			53
Total			653

#### 2.2.1 Boolean search

Boolean operators (AND/OR) were used to combine terms within and across these blocks. Because the indexing structure and search syntax differ between databases, the search expression was adapted to each source while preserving the same conceptual structure, Table 1. The mathematical terms included *“mathematical foundation,” “linear algebra,”* “calculus and optimization,” “probability and statistics,” *“information theory,” “graph theory,” “discrete mathematics,” “numerical analysis,” “stochastic mathematics,”* and “quantum mathematics and computing.” These terms were combined with AI-related terms, including *“artificial intelligence,” “machine learning,”* and *“deep learning,”* and healthcare-related terms, including *“healthcare,” “medicine,” “biomedical,” “radiology,”* and *“radiomics.”*

#### 2.2.2 Scopus and publish or perish search

The exact search combinations used in PoP (Google Scholar, PubMed/MEDLINE) are reported in Figure 3, Table 1 and Appendix A to ensure reproducibility. Search results were exported from Publish or Perish in RIS format and subsequently imported into VOSviewer v1.6.20 (Van Eck and Waltman, 2010) for bibliometric analysis. The principal Scopus search query is:

**Figure 3:**
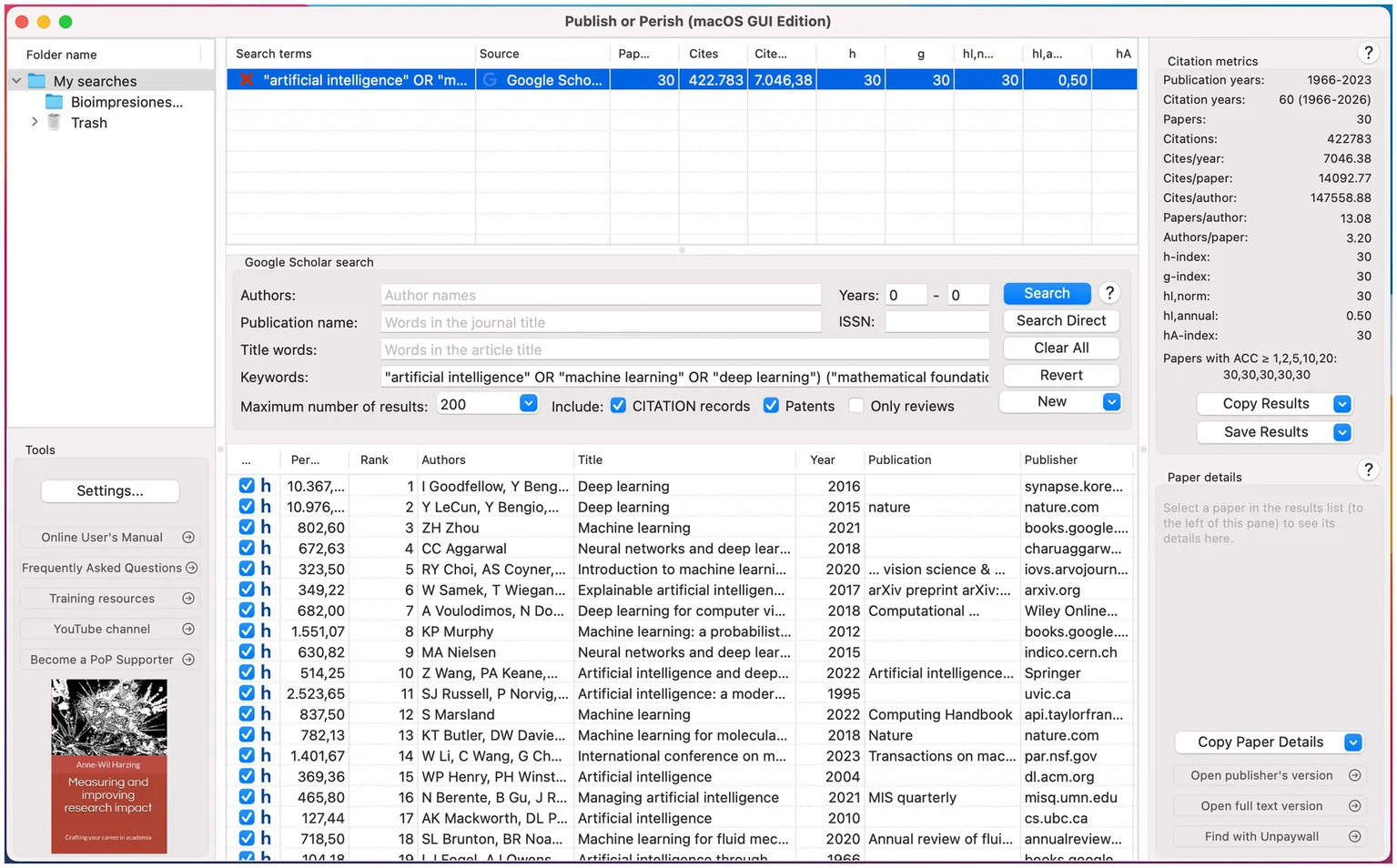
Publish or Perish (PoP) v8 interface showing the Google Scholar search strategy used to identify studies on the mathematical foundations of artificial intelligence in healthcare.

A standardized data-extraction form was used to systematically collect information from each eligible study. The extracted variables included author and publication year, study type, mathematical domain, mathematical concepts or equations, AI methodology or architecture, healthcare application, clinical task, dataset or data modality, validation approach, performance metrics, explainability or uncertainty methods, principal findings, methodological limitations, and bibliometric information.

### 2.3 Record deduplication

#### 2.3.1 Eligibility criteria

Studies were assessed using predefined inclusion and exclusion criteria. Eligible studies included:

##### 2.3.1.1 Inclusion criteria

- Peer-reviewed original research articles, systematic reviews, scholarly books, and book chapters addressing mathematical foundations underlying AI and their relevance to healthcare, including linear algebra, calculus, optimization, probability and statistics, information theory, graph theory, numerical analysis, stochastic processes, random matrix theory, and quantum computing.
- Studies developing or evaluating mathematical and computational foundations of AI methods, including machine learning, deep learning, generative AI, GNNs, explainable AI, uncertainty quantification, reinforcement learning, and quantum machine learning, with established relevance to healthcare.
- Studies applying AI algorithms, mathematical models, or computational frameworks to healthcare, including medical imaging, radiomics, bioinformatics, diagnosis, prognosis, clinical decision support, and precision medicine.
- Studies addressing trustworthy AI, including performance, robustness, calibration, uncertainty, reproducibility, explainability, fairness, transparency, safety, and clinical validation.
- Established ethical, reporting, governance, and methodological guidelines relevant to healthcare AI were used as supporting references rather than included in the quantitative synthesis.
- English-language publications from January 2016 to June 30, 2026 were included in the primary synthesis; earlier seminal works were retained only as foundational references when required to support key mathematical concepts.

##### 2.3.1.2 Exclusion criteria

- Conference abstracts, editorials, letters to the editor, commentaries, opinion papers, book reviews, dissertations, patents, and non-peer-reviewed publications.
- Studies unrelated to mathematical content, Artificial Intelligence, machine learning, deep learning, mathematical modeling, or healthcare applications.
- Articles with insufficient methodological information, incomplete data, or lacking a clear methodological or mathematical framework.
- Publications focused exclusively on software implementation or engineering aspects without discussing the underlying mathematical principles.
- Duplicate records identified across databases.
- Studies published before 2016.
- Articles published in languages other than English.

The systematic review corpus comprised exclusively the 147 studies that met the predefined eligibility criteria. Books, textbooks, theoretical references, methodological sources, and other background literature were used only to support conceptual or methodological statements and were not included in the PRISMA flow, study counts, quality assessment, VOSviewer bibliometric analyses, or quantitative synthesis.

###### 2.3.1.2.1 Application of eligibility criteria

Following duplicate removal, records were first screened by title and abstract for relevance to the intersection of mathematical foundations, artificial intelligence, and healthcare. Potentially eligible reports were then assessed in full text against the predefined inclusion and exclusion criteria. During full-text assessment, 54 reports were excluded based on their primary reason for ineligibility: not healthcare-related (*n* = 18), insufficient mathematical content (*n* = 12), wrong publication type (*n* = 10), outside the predefined publication date range (*n* = 8), non-English publication (*n* = 4), and other eligibility reasons (*n* = 2) such as insufficient methodological information/incomplete data and software-only/engineering studies. Each excluded report was assigned to one primary exclusion category to avoid double counting. The resulting 147 eligible studies constituted the final analytical corpus (Figure 3).

To avoid double-counting, findings from primary studies were counted only once in the descriptive synthesis; when the same primary study was discussed within an included review or systematic review, the review was used only for contextual or higher-level synthesis and did not contribute an additional independent observation.

### 2.4 Screening and study selection

Two reviewers independently screened all retrieved titles and abstracts according to the predefined eligibility criteria. Records considered potentially relevant by either reviewer proceeded to full-text assessment. The same reviewers independently evaluated the full-text articles for final eligibility (Appendix B). Disagreements at either stage were resolved through discussion and consensus. Reasons for full-text exclusion were documented. The complete selection process was summarized using a PRISMA 2020 (Page et al., 2021) flow diagram. Figure 4 details the workflow and the PRISMA methodology, respectively. Also, was registered in PROSPERO protocol (https://www.crd.york.ac.uk/PROSPERO/view/CRD420261487264, accessed on 24 August 2026).

**Figure 4:**
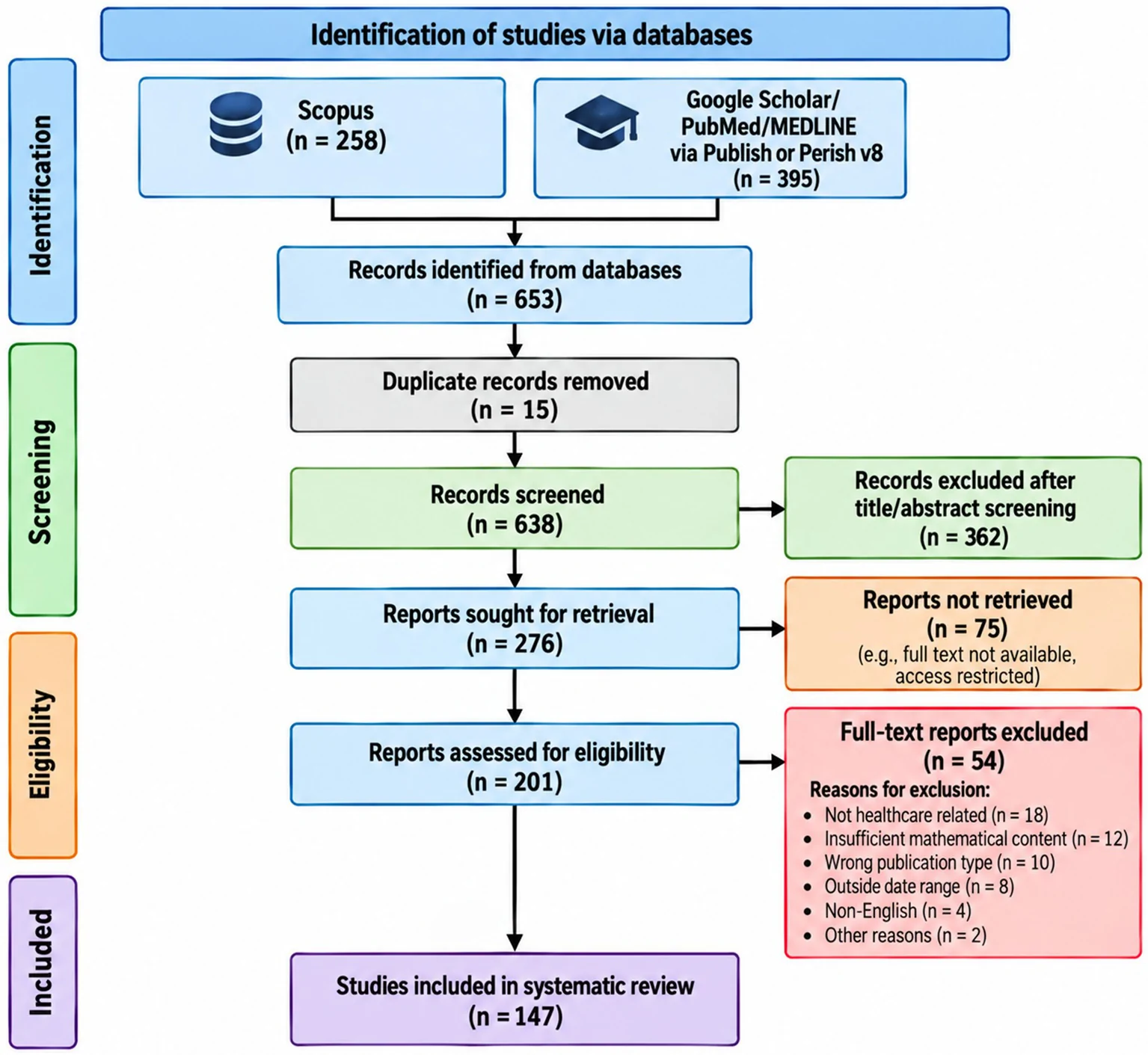
PRISMA flow diagram 2020 (Yepes-Nuñez et al., 2021) flow diagram template for systematic reviews. The new design is adapted from flow diagrams proposed by Boers (2018), Mayo-Wilson et al. (2018) and Stovold et al. (2014). Design by Biorender https://app.biorender.com.

### 2.5 Methodological quality assessment

Methodological quality was independently assessed by two reviewers using an adapted JBI-based critical appraisal framework designed to accommodate the heterogeneous study types included in this review (Appendices C–E). The framework comprised 12 domains: study design and objectives, population or data source, mathematical framework, AI/computational methods, data collection and measurement, bias and confounding, statistical analysis, model validation, reproducibility and transparency, results and reporting, interpretation and limitations, and clinical relevance and safety.

The criteria were applied according to study design. For empirical AI/clinical studies, emphasis was placed on data-source description, data acquisition, bias and confounding, statistical analysis, model validation, performance metrics, and clinical relevance. For methodological or computational studies, greater emphasis was placed on the mathematical framework, algorithmic description, parameterization, reproducibility, and validation. For theoretical/foundational studies, criteria requiring patient-level data, clinical measurements, or clinical validation were designated *not applicable (N/A)* rather than penalized. Review articles were assessed for methodological transparency, evidence synthesis, reproducibility, and appropriateness of their conclusions, while systematic reviews were additionally evaluated using AMSTAR-2 (Shea et al., 2017).

Each applicable criterion was rated as Yes (2 points), Partially/Unclear (1 point), or No (0 points); criteria considered N/A were excluded from the denominator. To permit comparison across study types with different numbers of applicable criteria, the total Quality Score (QS) was normalized as Equation 1:

$$\mathrm{QS}\;(\%)=\frac{\text{Score obtained}}{2\times k}\times 100$$ (1)

Where, *k* is the number of applicable criteria.

Studies were classified as high quality (≥80%), moderate quality (60–79%), or low quality (<60%). Representative study-level assessments are presented in Appendix C, and the complete operational appraisal criteria are provided in Appendix D.

Both reviewers completed the appraisal independently. Disagreements were resolved through discussion and consensus, with third-reviewer adjudication available when required. Inter-reviewer agreement was quantified using Cohen’s κ (κ = 0.89), indicating almost perfect agreement. Appendix E documents the reviewer-agreement and consensus procedure. Overall methodological quality and risk of bias were summarized using traffic-light plots. Confidence in the synthesized evidence was considered narratively according to methodological quality, consistency, validation adequacy, mathematical and statistical transparency, robustness, generalizability, and reproducibility. A formal GRADE assessment was not performed because of the substantial methodological and clinical heterogeneity and the predominantly non-interventional nature of the included evidence.

### 2.6 Evidence synthesis

A structured narrative and descriptive synthesis are performed for the 147 studies included in the systematic review. Owing to substantial methodological and clinical heterogeneity across mathematical approaches, AI architectures, healthcare applications, datasets, validation strategies, and outcome measures, a quantitative meta-analysis was not considered appropriate; therefore, the evidence was synthesized descriptively and comparatively.

Studies were categorized according to the principal mathematical foundations identified in the literature: (i) linear algebra; (ii) calculus and optimization; (iii) probability and statistics; (iv) information theory; (v) graph theory; (vi) discrete mathematics and numerical analysis; (vii) stochastic and advanced mathematical methods; and (viii) quantum mathematics and computing. Because individual AI approaches frequently rely on more than one mathematical foundation, domain coding was non-mutually exclusive, allowing a study to contribute to multiple mathematical categories when justified by its methodological content.

Within each mathematical domain, evidence was systematically compared according to study type, mathematical concepts and representative equations, AI methodology or architecture, healthcare application, validation approach, performance metrics, and principal findings. The synthesis focused on identifying recurring mathematical foundations, their functional roles within AI models, their relationships with specific healthcare applications, and emerging methodological trends.

Primary studies, methodological studies, and review articles were interpreted according to their respective study designs. To prevent overlap and double-counting of evidence, findings from primary studies were counted only once in the descriptive synthesis. When the same primary evidence was discussed within an included review or systematic review, the review was used for broader methodological or contextual synthesis and was not treated as an additional independent empirical observation. This approach enabled integration of heterogeneous evidence while maintaining a clear distinction between primary empirical findings and secondary evidence synthesis.

### 2.7 Bibliometric analysis

Bibliometric data from the final corpus file (147 records) exported from Publish or Perish in RIS format. *VOSviewer v1.6.20* (Van Eck and Waltman, 2010) was subsequently used for bibliometric mapping and visualization. In addition, the reference lists of eligible studies (Table 2) were screened to identify potentially relevant publications not retrieved through the primary electronic database searches. The dataset contained 459 unique authors and associated bibliographic metadata. Co-authorship analysis was performed using authors as the unit of analysis, with a minimum threshold of one document per author. Figures 5a–c present complementary bibliometric visualizations.

**Table 2:** Units of analysis for bibliometric analysis in VOSviewer.

Analysis type	Analysis unit
Co-authorship	Authors
	Organizations
	Countries
Co-occurrence	Keywords
	Author Keywords
	Index Keywords
Citation	Documents
	Sources
	Authors
	Organizations
	Countries
Bibliographic coupling	Documents
	Sources
	Authors
	Organizations
	Countries
Co-citation	Cited references
	Cited sources
	Cited authors

**Figure 5:**
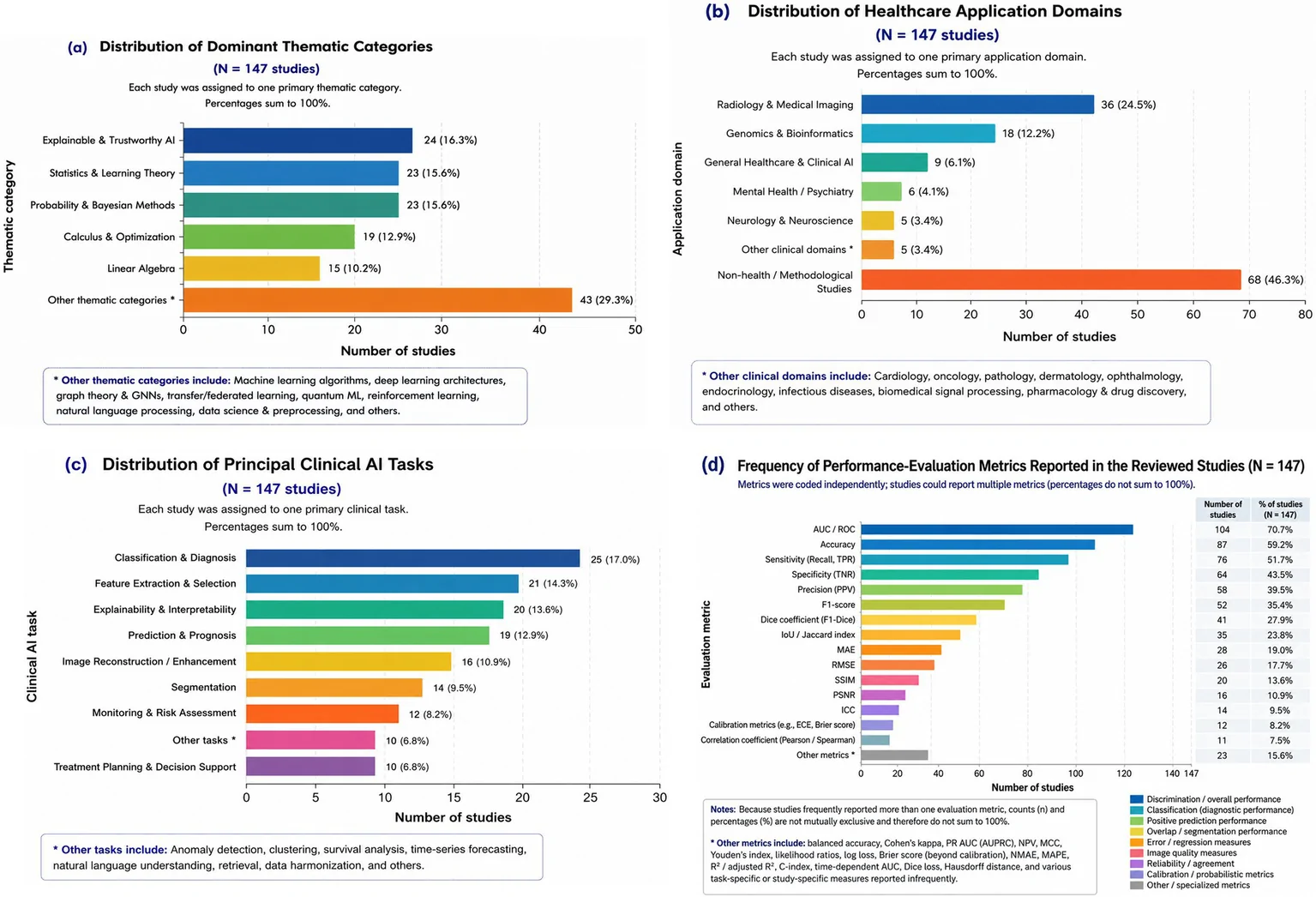
Distribution of thematic, clinical, and evaluation characteristics across the 147 studies included in the review. **(a)** Dominant thematic categories identified through content classification, with each study assigned to one primary category; **(b)** healthcare application domains represented in the reviewed literature; **(c)** principal clinical AI tasks addressed by the included studies; and **(d)** performance-evaluation metrics reported across the literature.

The parameters used for the bibliometric analyses are summarized in Table 1.

## 3 Results

A total of 147 studies met the eligibility criteria. Mathematical-domain coding was non-mutually exclusive because individual studies frequently incorporated multiple mathematical foundations. Mathematical-domain coding was non-mutually exclusive because individual studies frequently incorporated multiple mathematical foundations.

### 3.1 Mathematical domains on healthcare AI algorithms

AI systems combine mathematical concepts to learn from data and make decisions. Figure 6 and Table 1 shows different mathematical domains in AI and a simplified example of how mathematics works together in: (i) *Data Representation:* Linear algebra is used to represent data as vectors, matrices, and linear transformations. (ii) *Model Training*: Calculus and optimization algorithms adjust the model’s parameters to minimize errors essential for training AI models. Optimization is the process of finding the best solution to a problem, often by minimizing or maximizing a function. (iii) *Prediction:* Probability and statistics help the model make predictions and handle uncertainty, incomplete or noisy data. (iv) *Evaluation:* Statistical methods assess the model’s performance, provide tools for analyzing and interpreting data, which is crucial for training AI models and ensure it generalizes well to new data.

**Figure 6:**
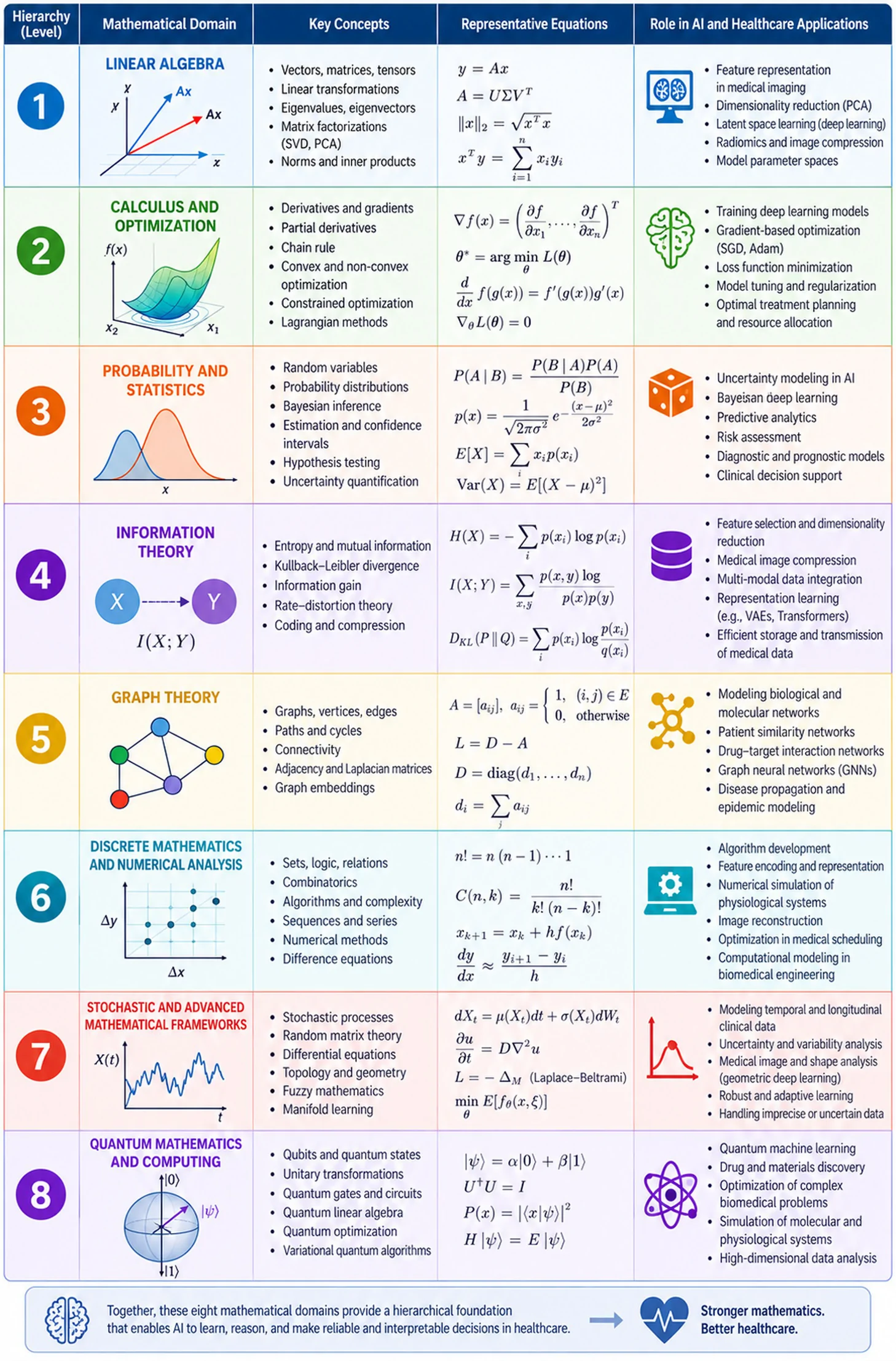
Hierarchical organization of the main mathematical domains underpinning artificial intelligence in healthcare, from foundational linear algebra and optimization to statistics, probability, discrete mathematics, numerical analysis, and graph theory, highlighting their key concepts, equations, and clinical applications.

#### 3.1.1 RQ1. Which mathematical domains most strongly underpin contemporary AI applications in healthcare?

For conceptual consistency, the mathematical foundations identified in this review were organized into eight overarching domains: (i) Linear algebra, (ii) Calculus and Optimization, (iii) Probability and Statistics, (iv) Information Theory, (v) Graph Theory, (vi) Discrete Mathematics and Numerical analysis, (vii) Stochastic and advanced mathematical, (viii) Quantum mathematics and computing. These domains were defined as an analytical taxonomy rather than as mutually exclusive categories because contemporary AI architectures frequently integrate multiple mathematical foundations within the same computational pipeline, see Tables 3–5. Table 3 presents mathematical domains, Table 4 presents primary empirical studies reporting original experimental or quantitative evidence, whereas Table 5 presents reviews, surveys, methodological studies, standardization frameworks, and theoretical/foundational contributions. Beyond these dominant domains, the review identified information theory, graph theory, discrete mathematics, numerical analysis, and quantum mathematics as complementary or emerging foundations.

**Table 3:** Core mathematical domains, formal objects, and enabling roles in artificial intelligence.

Domain	Principal objects	Representative equation	Role	Applications
Linear algebra	Vectors, matrices, tensors, affine transformations, eigendecomposition, SVD/PCA	h = φ(Wx + b); X = UΣVᵀ	Data representation, feature transformation, dimensionality reduction (CNNs, Transformers, DNNs, SVMs)	Radiomics, Imaging, Genomics, EHRs (Kim et al., 2025; Wu et al., 2021)
Calculus and optimization	Gradients, chain rule, loss minimization, convex optimization, Adam/SGD	θ* = arg min θ [L(θ) + λR(θ)] $\theta_{t+1}=\theta_{t}-\eta \nabla_{\theta }\;J(\theta )$	Parameter estimation and controlled model training (CNNs, U-Net, GANs, Transformers, SVMs) Gradient Descent.	Classification, segmentation, prognosis, reconstruction (Barakat and Bianchi, 2021)
Probability and statistics	Bayesian inference, likelihood, entropy, predictive variance, calibration	$p(\theta |D)=\frac{p(\theta |D)p(\theta )}{p(D)}$	Inference and uncertainty quantification (Bayesian ML, probabilistic DL, ensembles)	Diagnosis, prognosis, risk prediction, uncertainty-aware imaging (Kendall and Gal, 2017; Leibig et al., 2017)
Information theory Graph theory	Entropy, cross-entropy KL divergence, mutual information	H(x)=−∑p(x)logp(x)	Information measurement and distribution matching	Cross-entropy, VAE regularization, feature selection (Jiménez-Gaona et al., 2020; Wright and Recht, 2022)
	Graphs, adjacency matrices, Laplacians, combinatorial	L = D-A	Graph Laplacian, where D is the degree matrix and A is the adjacency matrix, (GNNs, graph convolution, knowledge graphs)	Classification, reconstruction, feature selection
Discrete mathematics and numerical analysis	Linear systems, inverse problems, iterative methods	minx ||Ax−b||^2^ + λ||Lx||^2^	Stable and scalable computation (CNN-based reconstruction, iterative ML, inverse-model AI)	CT/MRI Brain networks, molecular biology, EHRs, drug discovery
	Iterative solvers, inverse problems, conditioning, finite differences, low-rank approximations	R^n(f) = (1/n)ΣL(f(xᵢ),yᵢ)	SVMs, cross-validation, external validation	CT, MRI, PET, ultrasound, radiomics (Żądło and Chwila, 2025).
Stochastic and advanced mathematical	Stochastic processes, random matrix theory, topology, geometry, differential equations, fuzzy mathematics	L = D-A	Extends convolution and representation learning to non-Euclidean graph- and manifold-structured data.	Brain networks, genetic/regulatory networks, sensor networks, and 3D surfaces (Leibig et al., 2017).
Quantum mathematics and computing	Hilbert spaces, quantum states, kernels, variational circuits	${ℒ}_{\mathrm{SC}}=-\sum_{i}\log\frac{e^{z_{i}^{⊤}z_{j}/\tau }}{\sum_{k}e^{z_{i}^{⊤}z_{k}/\tau }}$	Supervised contrastive loss. QSVM, VQC, quantum CNNs, QML	Radiomics, cancer imaging, medical-image classification (Kottahachchi Kankanamge Don and Khalil, 2025; Wei et al., 2023)

*See Annex A for more statistical details.

**Table 4:** Primary empirical evidence linking mathematical foundations, AI methodologies, and healthcare applications.

Mathematical domain	Reference/study type	Mathematical concept	Mathematical formulation	AI architecture/function	Healthcare application/dataset	Main findings/quantitative evidence
Linear algebra	Kim et al. (2025)—Empirical	Matrix–vector multiplication; optical matrix–vector composition	y=Wx	Optical matrix–vector multiplication for encoding, decoding, feature projection, and neural-network inference.	Image reconstruction and accelerated AI computation.	Optical matrix–vector multiplication achieved reconstruction MSE = 0.0251–0.0547 and supported autoencoder, VAE, and GAN encoding/decoding tasks.
Calculus and optimization	Schott et al. (2024) Empirical	Chain rule; localized gradients; gradient-based uncertainty quantification	$\frac{\partial L}{\partial \theta_{i}}=\frac{\partial L}{\partial z}\frac{\partial z}{\partial \theta_{i}}$ $\nabla_{\phi }T_{R}$	Gradient-based sensitivity and localized uncertainty estimation.	Deep-learning medical-image assessment; uncertainty maps for clinically relevant image regions.	Local-gradient UQ showed greater sensitivity to image degradation (62.35% vs. 2.16%), improved false-positive filtering (+20.1% ROC-AUC; −26% FPR), and improved malignancy-related discrimination by +16.2% ROC-AUC.
Probability and statistics	Leibig et al. (2017) Empirical	MC predictive mean; epistemic uncertainty	$\bar{p}(y|x)=\frac{1}{T}\sum_{t=1}^{T}p_{t}(y|x)$ $\mathrm{Var}[p(y|x)]\approx \frac{1}{T}\sum_{t=1}^{T}{\left(p_{t}-\bar{p}\right)}^{2}$	Monte Carlo dropout for predictive uncertainty and referral of uncertain cases.	Diabetic-retinopathy screening.	Referring the 20% most uncertain cases increased retained-case accuracy from ~87% to ≥90% and supported performance above 85% sensitivity and 80% specificity.
	Kendall and Gal (2017) Empirical/methodological	Aleatoric and epistemic uncertainty	$\sigma_{a}^{2}(x)$	Learns observation-dependent noise and combines it with model uncertainty.	Image segmentation and depth estimation; methodological relevance to medical imaging.	Reported 67.5% mean IoU on CamVid and 70.6% accuracy/37.3% IoU on NYUv2; depth estimation achieved relative error = 0.110 and RMS = 0.506.
Statistical evaluation and validation	Jiménez-Gaona et al. (2024) Empirical	PSNR; SSIM; MSE; GAN image-quality evaluation	$\text{PSNR}=10\log_{10}(\frac{\mathrm{MA}X_{I}^{2}}{\mathrm{MSE}})$ $\text{SSIM}(x,y)=\frac{\left(2\mu_{x}\mu_{y}+C_{1})(2\sigma_{\mathrm{xy}}+C_{2}\right)}{\left(\mu_{x}^{2}+\mu_{y}^{2}+C_{1})(\sigma_{x}^{2}+\sigma_{y}^{2}+C_{2}\right)}$ $\mathrm{MSE}=\frac{1}{N}\sum_{i=1}^{N}{\left(y_{i}-{\hat{y}}_{i}\right)}^{2}$	Evaluation of synthetic/reconstructed images and downstream classification.	Breast ultrasound and mammography augmentation and classification.	SNGAN achieved FID = 52.89 for mammography and CGAN FID = 116.03 for ultrasound. ResNet18 without augmentation achieved 93.1%/94.9% benign/malignant ultrasound accuracy and 80.9%/76.9% mammography accuracy.
	Zhovannik et al. (2022) Empirical	Dice; segmentation uncertainty	$\text{Dice}=\frac{2\mathrm{TP}}{2\mathrm{TP}+\mathrm{FP}+\mathrm{FN}}$	Measures overlap and robustness to segmentation perturbations.	NSCLC radiomics; 421 training and 240 testing patients.	Prognostic stratification remained significant in 90% of 5,000 realizations for robust features versus ~30% for uncertainty-sensitive features; prediction uncertainty was 10% vs. 50%.

	Huynh et al. (2023) Empirical	Accuracy; AUC; MCC; sensitivity; specificity	$\text{Accuracy}=\frac{\mathrm{TP}+\mathrm{TN}}{\mathrm{TP}+\mathrm{TN}+\mathrm{FP}+\mathrm{FN}}$ $\text{Sensitivity}=\frac{\mathrm{TP}}{\mathrm{TP}+\mathrm{FN}}$ $\text{Specificity}=\frac{\mathrm{TN}}{\mathrm{TN}+\mathrm{FP}}$	Evaluation of CNN-based radiomics and multimodal predictive modeling.	Head-and-neck cancer PET/CT; disease-free survival (DFS) and overall survival (OS).	DFS: AUC = 0.67–0.75, scaled MCC = 0.65–0.72. OS: AUC up to 0.75; combined models reached approximately AUC = 0.80 and MCC = 0.70–0.77.
Fatima et al. (2024) Empirical	IoU/Jaccard; Dice; EF correlation; MAE	$\mathrm{IoU}=\frac{\mathrm{TP}}{\mathrm{TP}+\mathrm{FN}+\mathrm{FP}}$ $\text{Dice}=\frac{2\mathrm{TP}}{2\mathrm{TP}+\mathrm{FP}+\mathrm{FN}}$	Pix2Pix GAN with U-Net generator and PatchGAN discriminator for segmentation.	2-D echocardiography segmentation.	Dice = 0.961/0.930 (ED/ES, 2CH) and 0.959/0.950 (ED/ES, 4CH); EF correlations = 0.95 and 0.98; MAE = 3.2 and 2.1 mL.
Probability and statistics	Shoemaker et al. (2023) and Hersh et al. (2023) Empirical/methodological	Posterior feature-inclusion probability	$\mathrm{PP}I_{j}=P(\gamma_{j}=1|D)$	Bayesian ranking and selection of reliable radiomic biomarkers.	Oncology/radiomics; HPV case study.	Simulations: AUC = 0.986–0.991 and feature-selection MCC = 0.96. HPV case study: PPI > 0.50 selected 11/162 features (7%), sensitivity = 66.6%, specificity = 70%, Youden index = 0.37.
Quantum computing	Kottahachchi et al. (Kottahachchi Kankanamge Don and Khalil, 2025) Empirical	Supervised contrastive learning; VQC cross-entropy; parameterized quantum circuit	$L_{C}=-\frac{1}{N}\sum_{i=1}^{N}\sum_{j=1}^{C}p_{\mathrm{ij}}\log q_{\mathrm{ij}}$ $L_{\sup}=-\sum_{i}\log(\frac{\exp(z_{i}\cdot z_{p}/\tau )}{\sum_{a}\exp(z_{i}\cdot z_{a}/\tau )})$	Quantum representation learning followed by variational quantum classification.	Potential methodological relevance to biomedical image classification.	6-qubit VQC: accuracy 90% MNIST, 90% FMNIST, 75% KMNIST, 65% CIFAR10. SU2/AE/TL circuits: MNIST 90/80/95%; FMNIST 90/75/80%; KMNIST 75/60/55%.
	Huang et al. (2025)—Empirical	Quantum feature mapping; quantum-kernel similarity	$|\phi (x)\rangle =U_{\phi }(x)|0\rangle$ $K(x,z)={|\phi (x)|\phi (z)|}^{2}$	Maps radiomic features into Hilbert space and supplies kernel matrices for QSVC/QNN classification.	Pulmonary ground-glass-nodule classification and risk stratification from CT radiomics.	QNN: accuracy 89.23%, sensitivity 96.55%, specificity 83.33%, AUC = 0.937, OR = 2.536, *p* = 0.003; classical SVM: accuracy 76.56%, AUC = 0.833.
Quantum explainability/XAI	Pira and Ferrie (2024) Empirical/methodological	Q-LIME/local quantum-model explanation	$\xi (x)=\arg\min_{g\in G}\{L(f,,g,,\pi_{x})+\Omega (g)\}$	Local surrogate explanation of QNN predictions.	Quantum-model interpretability; potential bioinformatics and medical-imaging applications.	For an ambiguous test point, opposite explanations occurred ≈50% of the time and surrogate predictions disagreed with the QNN label ≈50% of the time, revealing a local region of model indecision.
	Meenakshi et al. (2025)—Empirical	Parameterized quantum circuit/quantum neural network	∣ψ(θ,x)〉=U(θ)U(x)∣0〉	Hybrid quantum–classical decision support with feature attribution and explainability.	Radiological decision support and medical-image classification.	Reported a 7.3% improvement in radiological detection sensitivity relative to conventional deep learning; quantum feature mapping was combined with classical classification and saliency/Shapley-based explanation.
Quantum computing	Simen et al. (2024)—Empirical	Digital–analog quantum convolution; quantum kernels; entanglement	$K(x,x^{\prime })={|\phi (x)|\phi (x^{\prime })|}^{2}$	Digital–analog quantum convolution and feature extraction (DAQCNNs).	Image classification; methodological relevance to biomedical imaging.	BreastMNIST: AUC = 0.926, ACC = 0.897 vs. classical CNN: AUC = 0.903, ACC = 0.885. PneumoniaMNIST: AUC = 0.963, ACC = 0.858 vs. classical CNN: AUC = 0.957, ACC = 0.849.

*NR (theoretical/review study; no single empirical performance estimate reported).

**Table 5:** Secondary, methodological, and foundational evidence supporting the mathematical framework of AI in healthcare.

Mathematical domain	Reference/study type	Mathematical concept	Mathematical formulation	AI architecture/function	Healthcare application/dataset	Main findings/quantitative evidence
Linear algebra	Hossen et al. (2026)	Convolution; pixel-wise softmax; weighted cross-entropy	$Y(i,j)=\sum_{m}\sum_{n}K(m,n)X(i-m,j-n)$ $p_{k}(x)=\frac{\exp(a_{k}(x))}{\sum_{j}\exp(a_{j}(x))}$ $L=-\sum_{x}w(x)\log p_{y(x)}(x)$	U-Net; encoder–decoder; skip connections; attention mechanisms; transformer-based U-Nets; Dice; IoU	Medical image segmentation across MRI, CT, dermoscopy, retinal imaging, microscopy.	U-Net variants generally report Dice ≈85–90%, attention-based models ≈90–95%, while selected transformer-based/advanced variants exceed 95%.
	Shamshad et al. (2023) Probability	Scaled dot-product self-attention; query-key-value projections; softmax normalization; positional representations	$\text{Attention}(Q,K,V)=\text{softmax}(QK^{T}/\surd d_{k})V$	Transformer/Vision Transformer. Computes global pairwise relationships between query and key representations and aggregates value vectors to capture long-range context.	Medical-image segmentation, detection, classification, reconstruction/restoration, synthesis, registration, and clinical report generation.	Comprehensive survey covering >125 papers on Transformers in medical imaging, showing that self-attention enables global contextual modeling beyond the local receptive fields of conventional CNNs.
Graph theory/linear algebra	Wu et al. (2021) Survey	Adjacency matrices; neighborhood aggregation; graph convolution; message passing; graph representation learning	$H^{\left(l+1\right)}=\sigma (D\sim^{-1/2}\;\tilde{A}{\tilde{D}}^{-1/2}H^{\left(1\right)}W^{\left(1\right)})$	GNNs learn node-, edge-, and graph-level representations by propagating and transforming information over non-Euclidean graph structures.	General graph applications with direct relevance to biomedical networks, molecular graphs, patient-similarity graphs, brain connectivity, and knowledge graphs.	Organizes GNNs into 4 major families: recurrent GNNs, convolutional GNNs, graph autoencoders, and spatial–temporal GNNs.
Probability and statistics	Abdar et al. (2021) Review	Bayesian inference; MC dropout; ensemble uncertainty	p(θ∣D)∝p(D∣θ)p(θ)	Predictive uncertainty estimation in deep learning.	Medical imaging and broader uncertainty-aware AI applications.	Identifies MC dropout and ensemble-based methods as major practical approaches for predictive uncertainty quantification. NR: review; no single pooled performance estimate.
Probability and statistics	Chakraborti et al. (2025) Methodological	Prediction-set coverage	P{Y∈C(X)}≥1−α	Provides individual prediction sets with explicit statistical coverage.	Personalized/precision AI.	Highlights population imbalance as a source of personalized uncertainty; 86% of genomic-study data were reported as originating from individuals of European ancestry.
Statistical evaluation and validation	Faghani et al. (2023) Review/illustrative	Predictive variance	$\mathrm{Var}(\hat{y})=\frac{1}{T}\sum_{t=1}^{T}{\left({\hat{y}}_{t}-\bar{y}\right)}^{2}$	Separates aleatoric and epistemic uncertainty for model evaluation and safety assessment.	Radiology predictions and clinician referral of unreliable cases.	Illustrative examples showed uncertainty increasing from 0.1 (in-distribution) to 0.8 (out-of-distribution), with epistemic uncertainty = 0.7; identical 70% predicted probabilities could correspond to 5% vs. 20% uncertainty.
Statistical evaluation and validation	Salvi et al. (2025) Review/methodological	Predictive entropy	$H(X)=-\sum_{i}p(x_{i})\log_{2}p(x_{i})$	Measures total classification uncertainty and supports confidence-aware AI.	Healthcare explainability and uncertainty; detection of shortcut/site-specific signals.	Discusses that a deep-learning model could identify hospital system of origin with >99.9% accuracy, illustrating exploitation of acquisition/site-specific confounders rather than clinically relevant signal.
Statistical learning theory	Weihs and Ickstadt (2018)—Theoretical	Empirical risk minimization (ERM); capacity control; generalization	${\hat{R}}_{n}(f)=\frac{1}{n}\sum_{i=1}^{n}L(f(x_{i}),y_{i})$	Controls empirical error and model capacity to support generalization.	Theoretical basis for classification, regression, radiomics, prognosis, and predictive modeling.	Establishes that minimizing training error alone does not guarantee generalization; model capacity must also be controlled.
Probability, statistics evaluation and validation	Zwanenburg et al. (2020) and van Timmeren et al. (2020)—Standardization/review	Intraclass correlation coefficient (ICC); radiomic reproducibility	$\mathrm{ICC}=\frac{\sigma_{b}^{2}}{\sigma_{b}^{2}+\sigma_{w}^{2}}$	Quantifies feature reliability and repeatability across readers, scanners, and segmentations.	Radiomics across MRI, PET, and CT.	IBSI standardized 169 radiomic features across 25 teams; 167/169 (98.82%) showed good-to-excellent reproducibility in the reported comparison. ICC is emphasized as a key robustness metric.
Graph theory	Zhang et al. (2019) Review	Graph convolution; neighborhood aggregation; cross-entropy for graph classification	$H^{\left(l+1\right)}=\sigma \;({\tilde{D}}^{-1/2}\tilde{A}{\tilde{D}}^{-1/2}H^{\left(l\right)}W^{\left(l\right)})$ $L=-\sum_{i}\sum_{c}y_{\mathrm{ic}}\log p_{\mathrm{ic}}$	GCN feature propagation and message passing for node/edge/graph learning.	Population graphs, biological networks, drug–target prediction, disease modeling.	GCNs learn graph representations through normalized neighborhood aggregation; the expressive power of standard neighborhood-aggregation GNNs is bounded by the 1-dimensional Weisfeiler–Lehman (1-WL) test. NR for a single clinical performance estimate.
Discrete mathematics and numerical analysis	Betcke and Schönlieb (2023) Methodological	Regularized inverse problems	$\min_{x}\{∥\mathrm{Ax}-b{∥}_{2}^{2}+\lambda ∥\mathrm{Lx}{∥}_{2}^{2}\}$	Stabilizes reconstruction from noisy or incomplete measurements.	CT, MRI, PET, optical imaging, denoising, compressed sensing.	Demonstrates the role of inverse-problem regularization in stable biomedical image reconstruction. NR for a single pooled performance estimate.
Discrete mathematics and numerical analysis	Demi (2018) Methodological	Delay-and-sum beamforming	$y(t)=\sum_{m=1}^{M}w_{m}x_{m}(t-\tau_{m})$	Phase/time-aligned signal summation for image reconstruction.	Ultrasound and echocardiography.	Demonstrates how delay-and-sum beamforming reconstructs ultrasound images by compensating propagation delays before signal summation. NR for a single universal performance estimate.
Quantum computing	Wei et al. (2023) Review	Born rule; Hilbert-space representation	$P(c|x)={|c|\psi (x)|}^{2}$ $|\psi \rangle =\sum_{i}\alpha_{i}|i\rangle ,\;\sum_{i}{|\alpha_{i}|}^{2}=1$	Maps quantum-state amplitudes to class probabilities; establishes quantum state representation for QML.	Quantum medical-image classification and quantum CNN concepts.	Establishes quantum-state representation and Born-rule measurement as mathematical foundations of QML for medical imaging. NR: survey; no single empirical performance estimate.
Quantum explainability/XAI	Ray et al. (2025) Review/mixed evidence	Shapley-value feature attribution	$\phi_{i}=\sum_{S\subseteq N\backslash \{i\}}\frac{|S|!(n-|S|-1)!}{n!}[f(S\cup \{i\})-f(S)]$	Global/local feature attribution for hybrid and quantum AI.	Precision medicine, biomarker discovery, medical imaging, and radiomic-feature interpretation	The review identifies Shapley-value and LIME-based approaches as relevant strategies for interpreting quantum and hybrid quantum–classical models in precision-medicine applications.
Quantum computing	Widdows et al. (2021)—Theoretical	Quantum states; density matrices; tensor-product representations	$\rho =\sum_{i}p_{i}|\psi_{i}\rangle \;\langle \psi_{i}|$	Mathematical representation of probabilistic and compositional information in quantum-inspired AI.	Information retrieval, NLP, semantic reasoning; conceptual relevance to healthcare AI.	Provides a common mathematical framework linking quantum theory and vector-space AI. NR: theoretical study; no single empirical performance estimate.
Quantum computing	Yan et al. (2024)—Review	Quantum image/state representation	$|\psi \rangle =\sum_{i=0}^{N-1}\alpha_{i}|i\rangle ,\;\sum_{i=0}^{N-1}{|\alpha_{i}|}^{2}=1$	Quantum-enabled image processing, classification, optimization, and image security.	Medical-image processing.	Disease diagnosis represented 48.89% and medical-image security 26.06% of the reviewed quantum-enabled applications.

Linear algebra was identified in approximately 100/147 studies (68.0%), calculus and optimization in 91/147 (61.9%), and probability and statistics in 79/147 (53.7%). The findings are presented according to the four predefined research questions and reflect the occurrence of each mathematical foundation across the corpus and are not expected to sum to 100%.

According, Table 3 *Linear algebra* primarily supported the representation and transformation of high-dimensional biomedical data through vectors, matrices, tensors, embeddings, matrix multiplication, convolution, eigen decomposition, and dimensionality-reduction techniques. These operations underpinned CNNs, U-Net architectures, deep neural networks (Jiménez-Gaona et al., 2020), Transformer-based models, radiomics pipelines, and other representation-learning approaches. Representative applications included medical-image classification, segmentation, reconstruction, genomics, and feature extraction.

The affine transformation h = ∅ (W_x + b) introduced in the deep learning framework of Goodfellow et al. (2016) and Bishop and Bishop (2024) remains the fundamental equation for neural representation learning because it defines how information propagates through every layer of a neural network. Equally important is the regularized optimization objective, described by Wright and Recht (2022), which establishes the theoretical basis for controlling overfitting while minimizing empirical loss. Meanwhile, the empirical risk minimization formulation of Żądło and Chwila (2025) provides the principal statistical framework for model generalization, whereas the graph Laplacian introduced by Bronstein et al. (2017) extends learning from Euclidean spaces to structured biomedical networks.

*Calculus and optimization* constituted the principal mathematical mechanisms for model learning. Gradient-based optimization, backpropagation, the chain rule, regularization, and adaptive optimization supported parameter estimation and loss minimization across CNNs, GANs, U-Nets (Hossen et al., 2026), Transformers (Shamshad et al., 2023), and related architectures. These methods were particularly prominent in classification, segmentation, reconstruction, and predictive modeling.

*Probability and statistics* formed the third major mathematical foundation (Ghayoumi, 2026), supporting inference, uncertainty quantification, model evaluation, calibration, and generalization. Bayesian inference, predictive distributions, Monte Carlo methods, statistical learning, and performance metrics were widely applied to diagnosis, prognosis, medical imaging, radiomics, and uncertainty-aware clinical decision support.

#### 3.1.2 RQ2. How are mathematical foundations associated with different AI architectures and healthcare applications?

The reviewed evidence demonstrated clear and systematic relationships between mathematical foundations, AI architectures, and healthcare applications. Rather than relying on a single mathematical discipline, healthcare AI is supported by an interconnected mathematical ecosystem in which linear algebra enables data representation, optimization drives model learning, and probability and statistics support inference, uncertainty quantification, and validation. Accordingly, most AI architectures do not exhibit a one-to-one correspondence with a specific mathematical domain; instead, they integrate multiple mathematical frameworks within the same computational pipeline.

*Linear algebra* was most strongly associated with deep neural networks, CNNs, U-Nets, Transformers, SVMs, and representation-learning methods. Matrix and tensor operations supported feature transformations and convolutional representations, while attention-based architectures extended these principles through vector embeddings and similarity operations.

In medical imaging, U-Net provided a representative range summarized in the review Hossen et al. (2026) report Dice ≈85–90%, attention-based models ≈90–95%, while selected transformer-based/advanced variants exceed 95%.

*Calculus and optimization* were closely associated with the training of CNNs, U-Nets, GANs, Transformers, and other differentiable architectures. Gradient-based learning and loss minimization enabled parameter estimation, whereas regularization and adaptive optimization contributed to convergence and generalization. Schott et al. (2024), for example, applied localized gradients to uncertainty quantification in deep-learning medical-image assessment and reported increased sensitivity to image degradation and improved discrimination of clinically relevant image regions.

*Probability and statistics* were particularly associated with Bayesian deep learning, uncertainty-aware neural networks, ensemble methods, radiomics, classification, prognosis, and model validation. Leibig et al. (2017) demonstrated the clinical relevance of uncertainty-aware prediction in diabetic-retinopathy screening: referring the 20% most uncertain cases increased retained-case accuracy from approximately 87% to at least 90%.

*Graph theory* and discrete mathematics were primarily associated with GNNs, graph convolution, message passing, population graphs, molecular networks, biomedical knowledge graphs, and drug–target modeling (Bronstein et al., 2017; Hamilton, 2020; Hogan et al., 2021; Abdar et al., 2021). These approaches extended healthcare AI from conventional Euclidean representations toward relational learning.

Numerical analysis was principally associated with inverse problems and computational reconstruction in CT, MRI, PET, ultrasound, and related imaging applications. Regularization, iterative methods, matrix conditioning, and numerical approximation contributed to stable reconstruction from noisy or incomplete biomedical measurements (Chakraborti et al., 2025).

Finally, *quantum mathematical* frameworks were associated with quantum neural networks (Kottahachchi Kankanamge Don and Khalil, 2025; Wei et al., 2023; Fronckova et al., 2018), variational quantum classifiers, quantum kernels, and hybrid quantum-classical models. Their healthcare applications remained concentrated primarily in radiomics, cancer-related classification, and medical-image analysis (Meenakshi et al., 2025; Yan et al., 2024; Simen et al., 2024).

Across clinical domains, radiology and medical imaging represented the largest specific healthcare application (24.3%), while classification and diagnosis constituted the most frequent clinical task (17.1%). The results therefore indicate that mathematical foundations are associated not only with particular algorithms but with specific stages of the healthcare-AI pipeline, from data representation and learning to prediction, reconstruction, validation, and interpretation.

However, Mathematical foundations operate as functional components of AI architectures: linear algebra enables representation, optimization enables learning, probability/statistics enable inference and validation, graph mathematics enables relational reasoning, and numerical methods enable stable reconstruction, with these functions combining differently according to architecture and clinical task.

#### 3.1.3 RQ3. How do classical and emerging mathematical frameworks contribute to explainability, uncertainty estimation, robustness, and clinical reliability?

The evidence showed that mathematical foundations contribute to trustworthy healthcare AI through several complementary mechanisms, particularly uncertainty quantification, statistical validation, explainability, robustness assessment, and reproducibility.

*Probability and statistics* provided the strongest direct mathematical basis for uncertainty estimation. Bayesian inference, Monte Carlo dropout, predictive variance, entropy, and ensemble-based approaches (Kendall and Gal, 2017; Schott et al., 2024; Abdar et al., 2021) enabled models to characterize uncertainty rather than return only deterministic predictions (Faghani et al., 2023). This distinction is clinically important because uncertainty-aware systems can identify cases requiring expert review or referral. Leibig et al. (2017), for example, demonstrated improved retained-case performance after referral of highly uncertain diabetic-retinopathy predictions.

Statistical frameworks also supported robustness and reproducibility assessment. The included studies used AUC/ROC, Dice, IoU, MCC, ICC, sensitivity, specificity, SSIM, PSNR, and related measures (Jiménez-Gaona et al., 2024) to evaluate different dimensions of model performance. Zwanenburg et al. (2020) demonstrated the importance of radiomic standardization, with 167 of 169 features (98.82%) showing good-to-excellent reproducibility in the reported multicenter comparison. Zhovannik et al. (2022) further demonstrated that segmentation uncertainty can propagate into downstream radiomic analyses, emphasizing the need to quantify variability rather than rely solely on average predictive performance.

*Explainability* represented another major component of trustworthy mathematical AI. The reviewed literature included feature-attribution methods, local surrogate explanations, uncertainty-aware explanations, interpretable modeling, and emerging quantum-XAI approaches (Ray et al., 2025; Ferrara, 2025; Fahim and Maji, 2025; Cohen and Huan, 2024). Collectively, these studies indicate that explainability alone is insufficient for clinical trustworthiness. Interpretability must be considered together with uncertainty, robustness, reproducibility, calibration, controllability, and ethical constraints.

Emerging mathematical frameworks provided additional mechanisms but exhibited different levels of maturity. Graph-based methods improved the representation of relational biomedical information, potentially increasing structural interpretability by explicitly representing relationships among patients, genes, molecules, or clinical concepts. However, their robustness depends strongly on graph construction and external validation.

Quantum approaches extended explainability (Guidotti et al., 2018; McDermid et al., 2021; Rudin, 2019) and representation into Hilbert-space and variational frameworks. Huang et al. (2025) reported QNN accuracy of 89.23%, sensitivity of 96.55%, specificity of 83.33%, and AUC of 0.937 for pulmonary ground-glass-nodule classification, compared with 76.56% accuracy and AUC of 0.833 for a classical SVM. Nevertheless, the limited number of clinically oriented studies and dependence on relatively small datasets or hybrid simulations prevent conclusions regarding general clinical superiority.

However, classical probability, statistics, optimization, and numerical methods currently provide the strongest mathematical support for uncertainty estimation, robustness, validation, and reproducibility, whereas graph-based, explainable, and quantum frameworks broaden the mechanisms available for interpretability and relational reasoning but require stronger clinical validation.

#### 3.1.4 RQ4. What methodological gaps and emerging mathematical directions currently shape the development of trustworthy and clinically translatable healthcare AI?

Several methodological gaps emerged from the synthesis. First, the literature showed substantial heterogeneity in evaluation and validation practices. AUC/ROC was the most frequently identified individual metric, but studies also reported accuracy, precision, sensitivity, specificity, Dice, IoU, MCC, ICC, SSIM, PSNR, uncertainty measures, and other task-specific metrics (Jiménez-Gaona et al., 2024). Although this diversity reflects differences among classification, segmentation, reconstruction, and prognostic applications, it limits direct cross-study comparison and demonstrates the absence of a standardized multidimensional evaluation framework.

Second, external and prospective clinical validation remained insufficient, particularly for emerging mathematical approaches. Many studies (Huynh et al., 2023; Fatima et al., 2024) demonstrated strong performance using retrospective, single-center, benchmark, or relatively limited datasets, whereas evidence from heterogeneous populations, multiple institutions, different acquisition systems, and prospective clinical workflows was less common.

Third, the reviewed literature (Jiménez-Gaona et al., 2020) showed that predictive performance remains more frequently evaluated than calibration, uncertainty, robustness, fairness, reproducibility, and clinical utility. High accuracy or AUC therefore cannot be assumed to imply clinical reliability. Future evaluation frameworks should integrate discrimination with calibration, uncertainty, robustness, subgroup performance, external generalization, and clinically meaningful outcomes.

Fourth, several emerging mathematical directions were identified. Graph neural networks constitute important mechanisms for relational learning in molecular, genomic, neurological, and patient-level data, but graph construction and interoperability remain methodological challenges. Quantum machine learning introduces Hilbert-space representations, quantum kernels, variational circuits, and quantum explainability, but currently remains predominantly proof-of-concept rather than clinically validated. The literature also points toward the reviewed literature in causal inference, topological methods, information-theoretic approaches, stochastic modeling, random matrix theory, and mathematically constrained learning, although these areas remain less represented than classical linear-algebraic, optimization, and probabilistic methods.

Additional thematic directions identified in the reviewed literature included large language models, federated learning, generative models, and other emerging AI approaches. These patterns highlight the presence of mathematical frameworks addressing not only predictive optimization but also reliability, interpretability, uncertainty, and clinical translation. The principal barrier is no longer simply the development of mathematically sophisticated AI models, but the lack of standardized, reproducible, externally validated, and clinically meaningful frameworks for demonstrating their trustworthiness and translational value.

Figure 5 summarizes the principal thematic, clinical, and evaluation characteristics of the 147 studies included in the review. Importantly, the four panels represent different analytical dimensions and should therefore be interpreted independently. Figure 5a presents dominant thematic categories, Figure 5b healthcare application domains, Figure 5c clinical AI tasks, and Figure 5d reported performance-evaluation metrics.

Figure 5a represents a dominant-theme classification. For the study-level analysis, a single publication could contribute to multiple mathematical domains for example, a deep-learning study could simultaneously involve linear algebra, calculus/optimization, and probability/statistics. Consequently, those domain-level percentages are not expected to sum to 100%. For Figure 6, however, each of the 147 studies was assigned to one primary thematic category according to its predominant methodological or conceptual focus. Explainable and trustworthy AI represented the largest of the explicitly reported categories, accounting for 24/147 studies (16.3%), followed by statistics and learning theory (23/147, 15.6%), probability and Bayesian methods (23/147, 15.6%), calculus and optimization (19/147, 12.9%), and linear algebra (15/147, 10.2%). The remaining thematic categories collectively represented 43/147 studies (29.3%). Thus, Figure 6 describes the relative prevalence of dominant research themes rather than the overall occurrence of mathematical foundations across individual studies.

Figure 5b characterizes the reviewed literature according to healthcare application domain. Radiology and medical imaging constituted the largest specific clinical application area, consistent with the extensive use of mathematically grounded AI methods for image classification, segmentation, reconstruction, radiomics, and computer-aided diagnosis. Other studies addressed genomics and bioinformatics, general healthcare and clinical AI, neurology and neuroscience, and additional clinical domains. A substantial component of the literature was primarily methodological or foundational rather than disease- or specialty-specific. This distinction is important because such studies contribute mathematical or computational methods applicable to healthcare AI without necessarily evaluating them directly in a clinical population.

Figure 5c presents the distribution of the principal AI tasks represented in the healthcare-oriented literature. Classification and diagnosis were the most prominent clinical tasks, followed by feature extraction and selection, explainability and interpretability, prediction and prognosis, and image reconstruction and enhancement. Collectively, these findings show that the mathematical foundations identified in the review support multiple stages of the AI pipeline: representation and feature construction, model optimization, diagnostic classification, predictive inference, image reconstruction, and interpretation of model outputs. The presence of explainability and interpretability as a substantial research theme further indicates is represented in the reviewed literature to the transparency and clinical interpretability of AI-assisted decisions.

Figure 5d summarizes the model-evaluation metrics reported in the reviewed literature. AUC/ROC was the most frequently identified individual metric, followed by precision, while accuracy and specialized measures such as the structural similarity index (SSIM) and intraclass correlation coefficient (ICC) occurred less frequently. These metrics address different aspects of model performance and should not be considered interchangeable: AUC/ROC evaluates discriminative ability across classification thresholds; precision quantifies the proportion of positive predictions that are correct; accuracy measures the overall proportion of correct classifications; SSIM evaluates structural similarity in image reconstruction or enhancement tasks; and ICC assesses agreement or reliability of quantitative measurements. The diversity of metrics therefore reflects the methodological heterogeneity of healthcare AI applications rather than a single standardized evaluation framework.

### 3.2 Explainability and trustworthy mathematical AI

Mathematical foundations play a central role in developing explainable and trustworthy AI (XAI) by providing formal mechanisms for interpreting model behavior, quantifying uncertainty, and supporting transparent decision-making. Ferrara (2025) highlights the emerging intersection between mathematics and XAI, emphasizing that mathematical structures are essential for understanding the mechanisms underlying AI predictions, while Fahim and Maji (2025) extend this perspective to generative AI, where mathematical frameworks support model formulation, optimization, and interpretation.

Beyond interpretability alone, Cohen and Huan (2024) integrate uncertainty-aware XAI into digital-twin systems, demonstrating the importance of jointly communicating predictions, explanations, and uncertainty to improve decision reliability. Guidotti et al. (2018) systematically categorize methods for explaining black-box models, including approaches for feature relevance, local and global explanations, rule extraction, and model approximation. However, explainability is not solely a technical problem: McDermid et al. (2021) emphasize its technical and ethical dimensions, particularly where AI-supported decisions have safety-critical consequences. In high-stakes settings, Rudin (2019) further argues that inherently interpretable models should be preferred over post-hoc explanations of opaque models whenever feasible.

Also, Ji et al. (2025) describes that AI systems behave consistently with human intentions and values. Its central contribution is identifying four fundamental objectives: Robustness, Interpretability, Controllability, and Ethicality. Trustworthy Mathematical AI section is that interpretability alone is insufficient. Trustworthy AI requires simultaneous consideration of robustness, interpretability, human controllability, and ethical behavior throughout the AI lifecycle. Technical assurance and governance as complementary mechanisms for managing risks.

### 3.3 VOSviewer mapping information

The final dataset, exported in RIS format, comprised 147 eligible records representing 459 authors. Bibliometric mapping was conducted to examine authorship patterns, collaborative networks, and the conceptual structure of the literature through co-authorship and keyword co-occurrence analyses. For the co-authorship analysis, authors were selected as the unit of analysis using the full-counting method. Documents with a large number of authors were restricted by setting the maximum number of authors per document to 25. The minimum number of documents per author was set to 1, resulting in 459 authors meeting the inclusion threshold. Network relatedness was normalized using the association-strength method, and the resulting author network was organized using the VOSviewer clustering algorithm.

For network visualization, layout optimization was performed using 1 random start, a maximum of 1,000 iterations, an initial step size of 1.00, a step-size reduction of 0.75, a step-size convergence threshold of 0.001, and a random seed of 0. For clustering, the advanced parameters were set to 10 random starts, 10 iterations, and a random seed of 0. These parameters were maintained consistently to improve the reproducibility of the bibliometric maps.

For the keyword co-occurrence analysis, keywords derived from the bibliographic records were used as the unit of analysis. A minimum occurrence threshold of 1 was applied. Of the 297 terms/keywords meeting this threshold, 99 were retained for visualization after relevance-based selection and data cleaning. Association-strength normalization was applied to quantify the relatedness between items. Where applicable, the analysis was examined using full and fractional counting to account for differences in how contributions and co-occurrences were weighted.

Before network construction, bibliographic data were cleaned to minimize terminological and author-name inconsistencies. The cleaning procedure included standardizing abbreviations and author-name variants, merging synonymous and singular/plural terms, and removing generic or non-informative terms. A VOSviewer thesaurus file was used to merge equivalent terms and exclude irrelevant terms. No manual modification of network links or cluster membership was performed after network generation.

These procedures were used to generate the bibliometric visualizations presented in Figure 7: (a) author co-authorship overlay visualization, (b) keyword co-occurrence density visualization, and (c) author co-authorship network visualization. Because each visualization represents a different aspect or representation of the bibliometric structure, the maps were interpreted accordingly.

**Figure 7:**
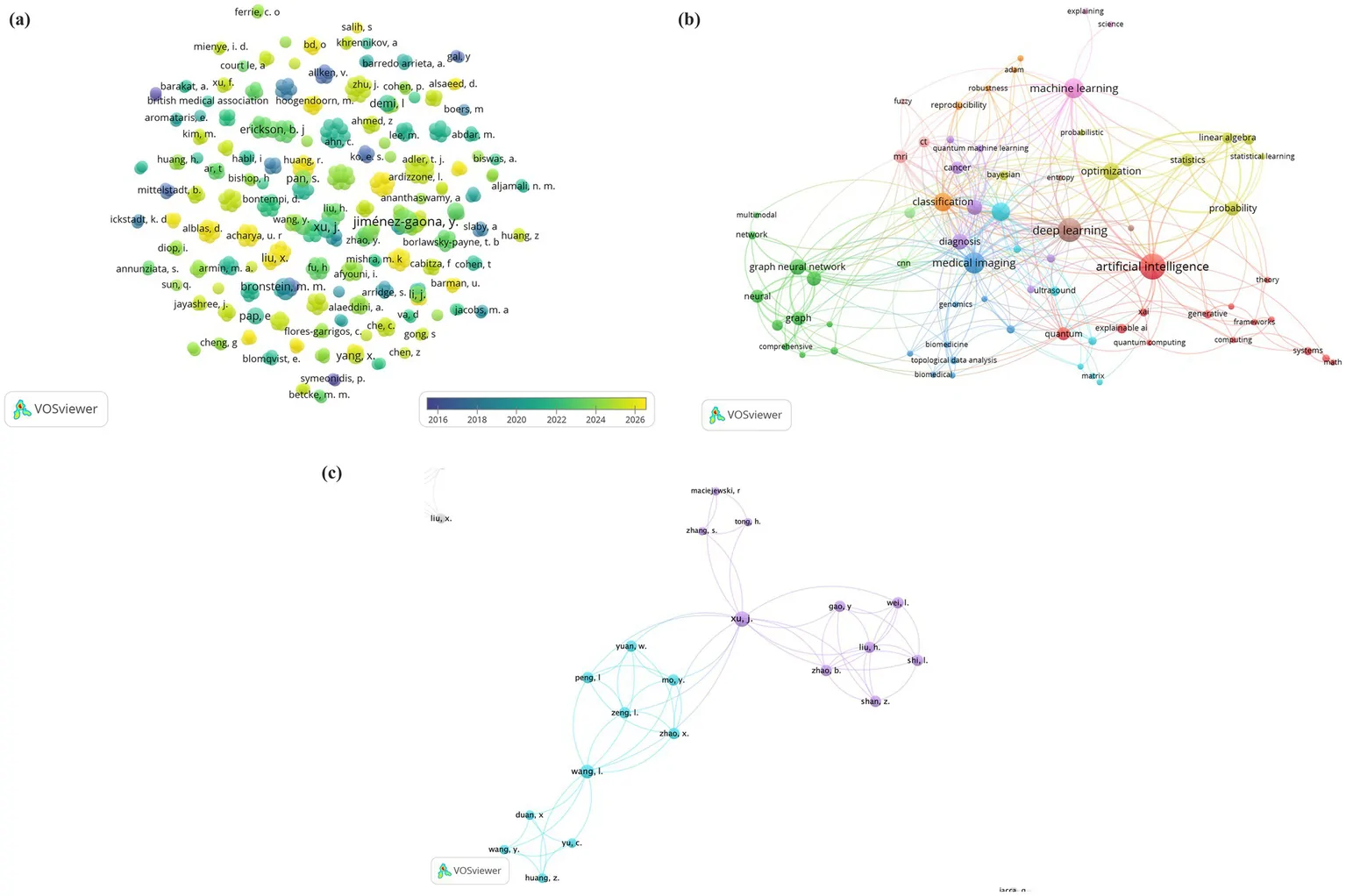
VOSviewer bibliometric mapping of the literature included in the systematic review. **(a)** Author co-authorship overlay visualization, in which nodes represent authors, links represent co-authorship relationships, and colors indicate the average publication year. **(b)** keyword co-occurrence density visualization, showing areas of higher concentration and connectivity among recurrent terms. **(c)** Author co-authorship network visualization, in which nodes represent authors, links indicate collaborative relationships, and colors identify clusters of interconnected authors.

Figure 7a presents the author co-authorship overlay visualization. Each node represents an author, and node size reflects the author’s relative weight within the network. Links between nodes represent co-authorship relationships, while the overlay color indicates the average publication year associated with each author according to the temporal scale displayed in the visualization. This map therefore combines information on scientific collaboration with the temporal distribution of contributing authors across the analyzed literature.

Figure 7b presents the keyword co-occurrence network derived from the bibliographic records of the included studies, illustrating the conceptual relationships among the principal mathematical foundations and AI-related topics represented in the analytical corpus. Node size reflects keyword occurrence frequency, links indicate co-occurrence relationships, and link thickness represents the relative strength of these relationships. Node colors identify clusters of closely related keywords generated by the VOSviewer clustering algorithm. The network shows connections among artificial intelligence and healthcare and mathematical domains including linear algebra, calculus and optimization, probability and statistics, graph theory, discrete mathematics and numerical analysis, stochastic and advanced mathematical methods, and quantum mathematics and computing. The visualization is interpreted as a structural representation of keyword relationships within the analyzed corpus rather than as evidence of temporal trends or changes in research importance.

Figure 7c shows the co-authorship network visualization. Each node represents an author, links indicate co-authorship relationships, and node size reflects the relative weight of the author within the network. Authors assigned to the same cluster represent more strongly interconnected collaborative structures according to the VOSviewer clustering algorithm. The network therefore enables identification of collaboration clusters as well as authors with fewer or weaker connections within the analyzed corpus.

Taken together, the three visualizations provide complementary perspectives on the bibliometric structure of the evidence base. The author overlay map characterizes collaboration in relation to publication time, the keyword density map identifies the principal conceptual concentrations and their relationships, and the co-authorship network reveals patterns of scientific collaboration. These findings complement the systematic synthesis by illustrating how authorship, research concepts, and collaborative relationships are organized across the literature on the mathematical foundations of AI in healthcare.

### 3.4 Methodological quality assessment

Methodological quality was assessed using the adapted JBI-based critical appraisal framework described in Section 2.6. Given the heterogeneity of the included evidence, the appraisal considered methodological rigor, data collection, mathematical and statistical analyses, AI methodology and validation, evaluation metrics, reporting transparency, and consistency between results and conclusions. Each applicable criterion was rated as Yes (2 points), Partially (1 point), or No (0 points). Scores were expressed as a percentage of the maximum applicable score to facilitate comparison across study designs and were classified as high (≥80%), moderate (60–79%), or low quality (<60%). Systematic reviews were additionally evaluated using AMSTAR-2.

Independent assessments by two reviewers demonstrated strong inter-reviewer agreement (Cohen’s κ = 0.89). Disagreements were resolved through discussion and consensus before final quality classification. Methodological quality and potential risk-of-bias concerns were summarized using traffic-light plots (Appendices D, E).

Overall, the included evidence was predominantly of moderate-to-high methodological quality. Healthcare-oriented studies, particularly those addressing radiomics, uncertainty quantification, and explainable AI, generally demonstrated stronger validation strategies and greater clinical applicability. In contrast, emerging approaches, including quantum machine learning and explainable quantum AI, showed promising methodological development but were limited by restricted reproducibility, small-scale or proof-of-concept evaluations, and insufficient large-scale clinical validation. These findings support the overall methodological credibility of the evidence base while highlighting the need for stronger reproducibility, external validation, and real-world clinical evaluation of emerging mathematical approaches.

## 4 Discussion

This systematic review shows that healthcare AI is supported by an interconnected mathematical ecosystem rather than by isolated computational techniques (Khan et al., 2024; Hasan et al., 2025; Mahmood et al., 2022; Annunziata and Treglia, 2023). Among the 147 included studies, linear algebra was represented in 68.0%, calculus and optimization in 61.9%, and probability and statistics in 53.7%, confirming that data representation, parameter optimization, statistical inference, and uncertainty estimation remain the dominant mathematical foundations of contemporary healthcare AI. Because mathematical-domain coding was non-mutually exclusive, these results also demonstrate that modern AI systems generally combine several mathematical frameworks within a single computational pipeline.

The predominance of linear algebra and optimization is consistent with the structure of modern deep-learning systems. Matrix and tensor operations provide the computational basis for neural representations, convolutions, embeddings, and attention mechanisms, whereas gradient-based optimization determines how model parameters are learned from data (Goodfellow et al., 2016; LeCun et al., 2015; Bishop and Bishop, 2024; Barakat and Bianchi, 2021; Wright and Recht, 2022). Their widespread use therefore reflects not only their theoretical importance but also their computational scalability. In healthcare applications, these foundations underpin image classification, segmentation, reconstruction, radiomics, multimodal modeling, and clinical prediction. The empirical studies synthesized in this review illustrate this relationship directly: U-Net (Wu et al., 2021) report Dice ≈85–90%, attention-based models ≈90–95% in biomedical segmentation, while adaptive and gradient-based approaches continue to provide the optimization mechanisms underlying current CNN, GAN, Transformer, and uncertainty-aware architectures.

A second major finding is the prominent role of probability, statistics, and uncertainty quantification in the reviewed literature. The reviewed literature encompasses approaches beyond point predictions toward models capable of indicating when their outputs may be unreliable. This shift is particularly important in healthcare, where a highly accurate model may still be unsafe if confidence estimates are poorly calibrated or if performance deteriorates under distribution shift. Leibig et al. (2017), for example, showed that referring the 20% most uncertain diabetic-retinopathy cases increased retained-case accuracy from approximately 87% to at least 90%, while uncertainty-aware approaches reviewed by Kendall and Gal (2017), Abdar et al. (2021), and Faghani et al. (2023) provide mathematical mechanisms for distinguishing epistemic and aleatoric uncertainty. These findings support a broader transition from performance-centered AI toward confidence-aware and risk-sensitive clinical decision support.

The review also highlights the importance of robust statistical validation. AUC/ROC was the most frequently reported individual evaluation metric, but the included literature used a wide range of complementary measures, including Dice, IoU, MCC, ICC, SSIM, PSNR, sensitivity, specificity, and predictive uncertainty. This diversity reflects genuine differences between classification, segmentation, reconstruction, and prognostic tasks; however, it also reveals a lack of standardization that complicates cross-study comparison. Importantly, high average performance does not necessarily imply reproducibility or clinical reliability. Zwanenburg et al. (2020), for instance, demonstrated the importance of standardization by evaluating 169 radiomic features across 25 teams, with 167/169 (98.82%) achieving good-to-excellent reproducibility in the reported comparison. Similarly, Zhovannik et al. (2022) showed that segmentation uncertainty can substantially alter downstream radiomic conclusions, reinforcing the need to evaluate variability rather than reporting only point estimates.

The thematic distribution further indicates that explainable and trustworthy AI has become a central research direction, representing approximately 16.3% of the dominant thematic classifications. This finding is consistent with growing recognition that interpretability alone is insufficient for high-stakes medical AI. Mathematical trustworthiness requires the integration of interpretability, uncertainty estimation, robustness, calibration, reproducibility, fairness, and controllability (Barredo Arrieta et al., 2020; Fahim and Maji, 2025; Fahim and Maji, 2025; Cohen and Huan, 2024; Guidotti et al., 2018; McDermid et al., 2021; Rudin, 2019). Rudin (2019) further argues that intrinsically interpretable models should be preferred to post-hoc explanations when feasible in high-stakes settings, while Ji et al. (2025) conceptualize trustworthy AI through the complementary dimensions of robustness, interpretability, controllability, and ethicality. Together, these perspectives suggest that explainability should be evaluated not as an isolated visualization procedure but as part of a broader mathematical and clinical validation framework.

Graph-based representations are particularly relevant for biomedical applications because conventional CNNs and dense neural networks primarily operate on Euclidean data, whereas biomedical information is often inherently relational and can be more naturally represented using graph structures. Graph neural networks therefore provide an appropriate mathematical structure for molecular interactions, brain connectivity, patient-similarity networks, drug–target relationships, and biomedical knowledge graphs (Bronstein et al., 2017; Hamilton, 2020; Hogan et al., 2021; Ghayoumi, 2026; Betcke and Schönlieb, 2023). These approaches extend conventional independent feature-vector representations by enabling relational reasoning among interconnected entities. Nevertheless, their clinical maturity remains lower than that of conventional deep-learning architectures because graph construction, node and edge definition, scalability, and validation across institutions remain highly problem-dependent.

The quantum-computing literature represents the most exploratory mathematical direction identified in this review. Quantum states, Hilbert spaces, quantum kernels, variational circuits, and quantum representations extend familiar linear-algebraic and probabilistic principles into non-classical computational frameworks (Kottahachchi Kankanamge Don and Khalil, 2025; Pira and Ferrie, 2024; Wei et al., 2023; Fatima et al., 2024; Weihs and Ickstadt, 2018; Salvi et al., 2025; van Timmeren et al., 2020). Some empirical results are encouraging. Huang et al. (2025) reported a QNN accuracy of 89.23%, sensitivity of 96.55%, specificity of 83.33%, and AUC of 0.937 for pulmonary ground-glass-nodule classification, compared with 76.56% accuracy and AUC of 0.833 for a classical SVM. However, such studies remain limited in number, frequently involve relatively small datasets or hybrid quantum-classical simulations, and cannot yet establish superiority over mature classical approaches. Quantum machine learning should therefore be regarded as an emerging research direction rather than a clinically validated technology.

The bibliometric findings complement the systematic synthesis by showing an interconnected field centered on deep learning, machine learning, artificial intelligence, medical imaging, computer-aided diagnosis, radiomics, and explainability (Lee et al., 2017; Wachter et al., 2017). Keywords, including large language models, federated learning, generative models, and bioinformatics suggest diversification beyond conventional image classification. However, the prominence of radiology and medical imaging (24.3% of healthcare applications) and classification/diagnosis (17.1% of clinical tasks) demonstrates that the available evidence remains concentrated in a subset of healthcare domains.

Overall, the principal contribution of this review is not the identification of a single “best” mathematical method, but the demonstration that trustworthy healthcare AI depends on the integration of complementary mathematical functions: linear algebra for representation, optimization for learning, probability and statistics for inference and uncertainty, numerical analysis for computational stability, graph theory for relational reasoning, and emerging quantum methods for alternative representations and computation. This integrated perspective extends previous specialized reviews (Gühring et al., 2022; Parekh and Jacobs, 2019; Barredo Arrieta et al., 2020; Wei et al., 2023) by connecting mathematical theory with AI architecture, validation strategy, healthcare application, and clinical trustworthiness within a single framework.

These findings reflect the rapid evolution of healthcare AI toward emerging paradigms such as generative AI, large language models (LLMs), reinforcement learning, and quantum computing. However, Shakeri and Far (2026) highlight that their clinical translation remains constrained by data quality, reliability, interpretability, ethical concerns, and regulatory readiness. These challenges underscore the need for rigorous mathematical frameworks supporting optimization, uncertainty quantification, robustness, and validation. Accordingly, clinical readiness should be demonstrated through reproducibility, external generalizability, calibrated uncertainty, and clinically meaningful performance rather than methodological sophistication alone.

Within this broader transition toward trustworthy AI, fairness represents an additional requirement for clinical translation. Recent evidence extends fairness beyond demographic equity to include dataset composition, model design, optimization strategies, and evaluation procedures. Shakeri et al. (2026) showed that data-splitting strategies, reward functions, and evaluation methods can introduce disparities across disease and molecular groups. Their framework emphasizes distributional and outcome parity, demonstrating that fairness should be quantitatively evaluated rather than treated solely as an ethical principle. Healthcare AI studies should therefore assess subgroup-specific performance and calibration, quantify potential disparities, and transparently report the mathematical criteria used to define and evaluate fairness.

These technical and fairness considerations should, in turn, be integrated with established ethical, reporting, and clinical evaluation frameworks. WHO guidance (World Health Organization, 2024) emphasizes governance, transparency, accountability, equity, and data protection, while TRIPOD+AI (Collins et al., 2024), DECIDE-AI (Vasey et al., 2022), and CONSORT-AI (Liu et al., 2020) provide guidance for transparent model reporting, early-stage clinical evaluation, and AI-based clinical trials, respectively. Collectively, these frameworks reinforce that trustworthy clinical translation depends not only on mathematical and computational performance but also on robust validation, transparent reporting, fairness assessment, ethical governance, and evaluation in real-world clinical settings.

A finally consideration is that algorithmic performance should be distinguished from clinical utility. High accuracy, AUC, sensitivity, specificity, or other predictive metrics demonstrate discriminatory or predictive capability under defined evaluation conditions, but do not by themselves establish improvements in patient outcomes, clinical decision-making, workflow efficiency, safety, or cost-effectiveness. Reyna et al. (2022) emphasize that conventional performance metrics may inadequately reflect clinical context and decision-making, contributing to the gap between high-performing algorithms and their adoption in practice. Similarly, recent translational evidence indicates that strong retrospective performance does not establish clinical readiness and that prospective evaluation, external validation, workflow integration, safety assessment, and real-world effectiveness are necessary to determine whether an AI system provides meaningful clinical value (Habibzadehomran, 2026; Morfaki, 2026; Reyna et al., 2022; Innocent et al., 2026). Therefore, healthcare AI should be evaluated across a continuum from technical performance to clinical utility and patient-centered outcomes, with algorithmic metrics regarded as necessary but insufficient evidence of clinical benefit.

### 4.1 Strengths and limitations

A major strength of this review is its integration of systematic evidence synthesis, mathematical characterization, methodological quality assessment, and bibliometric mapping across 147 studies. The review also distinguishes foundational or theoretical contributions from empirical studies and links mathematical formulations with their AI functions and healthcare applications. Independent methodological assessment by two reviewers, with Cohen’s κ = 0.89, further supports the consistency of the appraisal process.

Several limitations should nevertheless be considered. First, the literature search was restricted to Scopus and Google Scholar through Publish or Perish and to English-language publications, which may have excluded relevant studies indexed elsewhere or published in other languages. Second, Google Scholar has broad coverage but less standardized indexing than curated bibliographic databases, which may introduce variability in record retrieval and metadata quality. Third, the included literature was highly heterogeneous with respect to mathematical domains, AI architectures, healthcare applications, datasets, validation strategies, and outcome measures; therefore, a conventional meta-analysis was not appropriate, and the synthesis remained primarily narrative and descriptive.

Fourth, mathematical-domain coding was necessarily interpretative and non-mutually exclusive because individual studies frequently incorporated several mathematical foundations. Similarly, assigning one dominant theme for bibliometric or descriptive classification simplifies multidimensional studies and may underestimate conceptual overlap. Fifth, citation-based bibliometric measures are affected by publication-age bias, whereby older studies have had more time to accumulate citations than recent work. Network results may also depend on VOSviewer thresholds, normalization, and author/keyword cleaning procedures.

Sixth, the evidence base combines empirical studies, methodological papers, reviews, and foundational theoretical works. These sources contribute different forms of evidence and cannot be interpreted as equivalent demonstrations of clinical effectiveness. In particular, several emerging areas including quantum machine learning, explainable quantum AI, advanced graph learning, and uncertainty-aware systems remain supported mainly by methodological studies, simulations, or relatively limited clinical datasets.

The research evaluates the mathematical and methodological foundations of healthcare AI rather than the clinical effectiveness of AI interventions. Therefore, strong algorithmic performance should not be interpreted as evidence of improved patient outcomes, workflow efficiency, safety, or cost-effectiveness without prospective clinical evaluation.

Although the search incorporated multidisciplinary, biomedical, and technical literature sources, including Scopus, Google Scholar, PubMed/MEDLINE, some relevant studies may not have been retrieved because of differences in database indexing, search functionality, terminology, and coverage. This limitation was partially mitigated by screening the reference lists of eligible studies for additional potentially relevant publications.

## 5 Conclusion

This systematic review demonstrates that modern healthcare AI is built on a small number of highly recurrent but complementary mathematical foundations. Linear algebra (68.0%), calculus and optimization (61.9%), and probability and statistics (53.7%) were the most frequently represented domains, supporting data representation, neural-network learning, parameter optimization, statistical inference, uncertainty quantification, and model validation. These foundations are not used independently; rather, they interact throughout the complete AI pipeline.

A second major finding is the substantial representation of approaches incorporating uncertainty quantification, explainability, robustness, reproducibility, and trustworthy decision support alongside predictive performance. These findings are particularly important in healthcare, where reliable deployment requires more than high accuracy or AUC.

Graph-based learning and quantum machine learning extend the mathematical scope of healthcare AI toward relational and non-classical representations. However, their evidence base remains less mature than that of conventional deep-learning methods and requires stronger external, multicenter, and prospective validation.

Overall, the evidence indicates that mathematical rigor is an important component of the methodological foundations supporting reliability and interpretability of healthcare AI. Future clinically deployable systems will require transparent mathematical formulation, reproducible validation, calibrated uncertainty, robust external testing, and explicit evaluation of clinically meaningful outcomes rather than predictive performance alone.

### 5.1 Implications and future research

Future research should prioritize external and multicenter validation using heterogeneous populations, imaging systems, institutions, and acquisition protocols. Prospective studies should determine whether mathematically sophisticated AI systems improve clinical decisions, workflow efficiency, safety, or patient outcomes compared with established clinical and computational baselines.

Model evaluation should move beyond accuracy-centered reporting toward a multidimensional framework incorporating discrimination, calibration, uncertainty, robustness, subgroup performance, fairness, reproducibility, and external generalization. Reporting standards should also require transparent specification of mathematical choices, including loss functions, optimization strategies, regularization, uncertainty models, graph construction, feature representations, and validation procedures.

Trustworthy healthcare AI should increasingly integrate prediction, uncertainty, and explanation within the same model-evaluation framework. Clinicians need not only a predicted class or risk score but also an indication of confidence, relevant explanatory factors, and conditions under which the model may fail.

Emerging mathematical areas deserve targeted investigation. Graph neural networks require multicenter evaluation of graph-construction strategies and robustness to heterogeneous clinical relationships. Quantum machine-learning studies should progress from proof-of-concept benchmarks toward clinically relevant datasets, explicit classical baselines, hardware-aware validation, and reproducible quantum experiments. Additional research into causal inference, topological data analysis, information geometry, random matrix theory, and mathematically constrained learning may further improve generalization and interpretability in high-dimensional biomedical data.

Finally, reproducibility should become a central criterion for healthcare AI research through open datasets where ethically feasible, transparent preprocessing, standardized mathematical formulations, accessible implementations, predefined validation protocols, calibration analysis, subgroup evaluation, and clinically meaningful endpoints. Progress toward safe and trustworthy healthcare AI will ultimately require sustained collaboration among mathematicians, statisticians, clinicians, computer scientists, engineers, and ethicists.

## Data Availability

The original contributions presented in the study are included in the article/supplementary material, further inquiries can be directed to the corresponding author.

## Glossary

AI: artificial intelligence
AUC: area under the ROC curve
CNN: convolutional neural network
DFS: disease-free survival
ED/ES: end-diastole/end-systole
EF: ejection fraction
EHR: electronic health record
FID: Fréchet inception distance
FPR: false-positive rate
GCN: graph convolutional network
ICC: intraclass correlation coefficient
IoU: intersection over union
MCC: Matthews correlation coefficient
MRI: magnetic resonance imaging
MSE: mean squared error
OS: overall survival
PET: positron emission tomography
PQC: parameterized quantum circuit
PSNR: peak signal-to-noise ratio
QNN: quantum neural network
QSVC: quantum support vector classifier
SSIM: structural similarity index
UQ: uncertainty quantification
VAE: variational autoencoder
VQC: variational quantum classifier

## References

[ref1] Abdar M. Pourpanah F. Hussain S. Rezazadegan D. Liu L. Ghavamzadeh M. . (2021). A review of uncertainty quantification in deep learning: techniques, applications and challenges. Inf. Fusion 76, 243–297. doi: 10.1016/j.inffus.2021.05.008

[ref2] Ananthaswamy A. (2024). Why Machines Learn: The Elegant Math Behind Modern AI. New York: Penguin.

[ref3] Annunziata S. Treglia G. (2023). Editorial: Radiomics and artificial intelligence in radiology and nuclear medicine. Front. Med. 10:1216434. doi: 10.3389/fmed.2023.1216434, 37287746 PMC10242181

[ref5] Aromataris E. Lockwood C. Porritt K. Pilla B. Jordan Z., editors. JBI Manual for Evidence Synthesis. Adelaide, Australia: JBI (2024). Available online at: https://synthesismanual.jbi.global (accessed May 9, 2026).

[ref6] Barakat A. Bianchi P. (2021). Convergence and dynamical behavior of the ADAM algorithm for nonconvex stochastic optimization. SIAM J. Optim. 31, 244–274. doi: 10.1137/19m1263443

[ref7] Barredo Arrieta A. Díaz-Rodríguez N. Del Ser J. Bennetot A. Tabik S. Barbado A. . (2020). Explainable artificial intelligence (XAI): concepts, taxonomies, opportunities and challenges toward responsible AI. Inf. Fusion 58, 82–115. doi: 10.1016/j.inffus.2019.12.012

[ref8] Betcke M. M. Schönlieb C. B. (2023). Mathematics of biomedical imaging today—a perspective. Progress Biomed. Eng. 5:043002. doi: 10.1088/2516-1091/acd973

[ref9] Bishop C. M. Bishop H. (2024). Deep Learning: Foundations and Concepts, vol. 1 Cham, Switzerland: Springer.

[ref10] Boers M. (2018). Graphics and statistics for cardiology: designing effective tables for presentation and publication. Heart 104, 192–200. doi: 10.1136/heartjnl-2017-311581, 29030423

[ref11] Bronstein M. M. Bruna J. LeCun Y. Szlam A. Vandergheynst P. (2017). Geometric deep learning: going beyond Euclidean data. IEEE Signal Process. Mag. 34, 18–42. doi: 10.1109/MSP.2017.2693418

[ref12] Chakraborti T. Saria S. Subbaswamy A. Wiens J. (2025). Personalized uncertainty quantification in artificial intelligence. Nat. Mach. Intell. 7, 1–10. doi: 10.1038/s42256-025-01024-8

[ref13] Cohen P. Huan J. (2024). Uncertainty-aware explainable artificial intelligence as a foundational paradigm for digital twins. Front. Mech. Eng. 9:1329146. doi: 10.3389/fmech.2023.1329146

[ref14] Collins G. S. Moons K. G. Dhiman P. Riley R. D. Beam A. L. Van Calster B. . (2024). TRIPOD+ AI statement: updated guidance for reporting clinical prediction models that use regression or machine learning methods. BMJ, 385:e078378. doi: 10.1136/bmj-2023-078378 PMC11019967 38626948

[ref15] Demi L. (2018). Practical guide to ultrasound beam forming: beam pattern and image reconstruction analysis. Appl. Sci. 8:1544. doi: 10.3390/app8091544

[ref16] Faghani S. Moassefi M. Rouzrokh P. Khosravi B. Baffour F. I. Ringler M. D. . (2023). Quantifying uncertainty in deep learning of radiologic images. Radiology 308:e222217. doi: 10.1148/radiol.222217, 37526541

[ref17] Fahim Y. Maji S. K. (2025). “Mathematical frameworks for generative AI applications,” in Generative artificial Intelligence (AI) Approaches for Industrial Applications, (Cham: Springer Nature Switzerland), 47–67.

[ref18] Fatima N. Afrakhteh S. Iacca G. Demi L. (2024). Automatic segmentation of 2-D echocardiography ultrasound images by means of generative adversarial network. IEEE Trans. Ultrason. Ferroelectr. Freq. Control 71, 1552–1564. doi: 10.1109/TUFFC.2024.3393026, 38656835

[ref19] Ferrara M. (2025). Explainable artificial intelligence and mathematics: what lies behind? Let us focus on this new research field. Eur. Math. Soc. Mag. 135, 39–44. doi: 10.4171/mag/235, 42143461

[ref20] Fronckova K. Prazak P. Slaby A. (2018). “Singular value decomposition in image compression and blurred image restoration,” in Image Analysis and Recognition, eds. Campilho A. Karray F. ter Haar Romeny B., vol. 10882 (Cham: Springer).

[ref21] Ghayoumi M. (2026). Mathematical Foundations for Deep Learning. Boca Raton, FL: CRC Press, Taylor & Francis Group. doi: 10.1201/9781032690742

[ref22] Goodfellow I. Bengio Y. Courville A. (2016). Deep Learning. Cambridge, MA: MIT Press. Available online at: https://www.deeplearningbook.org (accessed May 9, 2026).

[ref23] Gühring I. Raslan M. Kutyniok G. (2022). Expressivity of deep neural networks. In Grohs P. Kutyniok G. (Eds.), Mathematical Aspects of Deep Learning. (Cambridge, UK: Cambridge University Press), 149–199. doi: 10.1017/9781009025096.004

[ref24] Guidotti R. Monreale A. Ruggieri S. Turini F. Giannotti F. Pedreschi D. (2018). A survey of methods for explaining black box models. ACM Comput. Surv. 51, 1–42. doi: 10.1145/3236009

[ref25] Habibzadehomran V. (2026). A responsible artificial intelligence framework for translational readiness of machine learning biomarker models in clinical decision support. Discov. Artif. Intell. 6:957. doi: 10.1007/s44163-026-02035-z

[ref26] Hamilton W. L. (2020). Graph Representation Learning. Synthesis Lectures on Artificial Intelligence and Machine Learning, San Rafael, CA: Morgan & Claypool Publishers. 14, 1–159.

[ref27] Harzing A. W. (2007). Publish or Perish (Version 8) [Computer Software] Tarma Software Research Pty Ltd. Available online at: https://harzing.com/resources/publish-or-perish

[ref28] Hasan M. Yasmin F. Fatima S. (2025). Advances in Deep Learning for Medical Imaging: Foundations, Evolution, and Impact. (Preprint). doi: 10.22541/au.176581998.82900297/v1

[ref29] Hersh W. R. Hoyt R. E. Chamberlin S. Ancker J. S. Gupta A. Borlawsky-Payne T. B. (2023). Beyond mathematics, statistics, and programming: data science, machine learning, and artificial intelligence competencies and curricula for clinicians, informaticians, science journalists, and researchers. Health Systems 12, 255–263. doi: 10.1080/20476965.2023.2237745, 37860593 PMC10583607

[ref30] Hogan A. Blomqvist E. Cochez M. d'Amato C. de Melo G. Gutierrez C. . (2021). Knowledge graphs. ACM Comput. Surv. 54:71, 1–37. doi: 10.1145/3447772

[ref31] Hossen M. F. Sarkar A. Majumder T. Nabi M. F. Ahamed M. F. Shafi F. B. . (2026). A comprehensive review of U-net architectures for medical image segmentation: emerging trends and federated learning perspectives. Neural Comput. & Applic. 38:493.

[ref32] Huang X. Xu F. Zhu W. Yao L. He J. Su J. . (2025). An integrated strategy based on radiomics and quantum machine learning: diagnosis and clinical interpretation of pulmonary ground-glass nodules. BMC Med. Imaging 25:279. doi: 10.1186/s12880-025-01813-y, 40646489 PMC12247411

[ref33] Huynh B. N. Groendahl A. R. Tomic O. Liland K. H. Knudtsen I. S. Hoebers F. . (2023). Head and neck cancer treatment outcome prediction: a comparison between machine learning with conventional radiomics features and deep learning radiomics. Front. Med. (Lausanne). 10:1217037. doi: 10.3389/fmed.2023.1217037 PMC10498924 37711738

[ref34] Innocent D. C. Anyakorah P. E. Innocent R. C. Dozie I. N. S. Chukwuocha U. M. Emerole C. O. (2026). Performance and clinical utility of image-based machine learning models for the diagnosis of neglected tropical diseases in low- and middle-income countries: a systematic review. BMC Infect. Dis. 26:1555. doi: 10.1186/s12879-026-13784-8, 42298484 PMC13505083

[ref35] Ji Y. Wei J. Zhang Y. Liu H. Wang X. Li B. (2025). AI alignment: a contemporary survey. ACM Comput. Surv. 58:132. doi: 10.1145/3770749

[ref36] Jiménez-Gaona Y. Álvarez M. J. R. Castillo-Malla D. García-Jaen S. Carrión-Figueroa D. Corral-Domínguez P. . (2024). BraNet: a mobil application for breast image classification based on deep learning algorithms. Med. Biol. Eng. Comput. 62, 2737–2756. doi: 10.1007/s11517-024-03084-1, 38693328 PMC11330402

[ref37] Jiménez-Gaona Y. Carrión-Figueroa D. Lakshminarayanan V. Rodríguez-Álvarez M. J. (2024). Gan-based data augmentation to improve breast ultrasound and mammography mass classification. Biomed. Signal Process. Control 94:106255. doi: 10.1016/j.bspc.2024.106255

[ref38] Jiménez-Gaona Y. Rodríguez-Álvarez M. J. Lakshminarayanan V. (2020). Deep-learning-based computer-aided systems for breast cancer imaging: a critical review. Appl. Sci. 10:8298. doi: 10.3390/app10228298

[ref39] Kaur R. Sharma D. (2025). “Application and significance of mathematics in artificial intelligence,” in Soft Computing and Signal Processing. ICSCSP 2024. Lecture Notes in Networks and Systems, eds. Reddy V. S. Wang J. Chetti P. Reddy K. T. V., vol. 1221 (Singapore: Springer).

[ref40] Kendall A., and Gal Y. (2017). What uncertainties do we need in Bayesian deep learning for computer vision? Advances in Neural Information Processing Systems, 30, 5574–5584. doi: 10.48550/arXiv.1703.04977

[ref41] Khan I. U. Ouaissa M. Ouaissa M. Fayaz M. Ullah R. (2024). Artificial Intelligence for Intelligent Systems: Fundamentals, Challenges, and Applications (1st ed.). Boca Raton, FL: CRC Press. doi: 10.1201/9781003496410

[ref42] Kim M. Kim Y. Park W. I. (2025). Image processing with optical matrix vector multipliers implemented for encoding and decoding tasks. Light Sci. Appl. 14:248. doi: 10.1038/s41377-025-01904-z, 40695802 PMC12284195

[ref43] Kottahachchi Kankanamge Don A. Khalil I. (2025). QRLaXAI: quantum representation learning and explainable AI. Quantum Mach. Intell. 7:24. doi: 10.1007/s42484-025-00253-9

[ref44] LeCun Y. Bengio Y. Hinton G. (2015). Deep learning. Nature 521, 436–444. doi: 10.1038/nature14539, 26017442

[ref45] Lee G. Lee H. Y. Ko E. S. Jeong W. K. (2017). Radiomics and imaging genomics in precision medicine. Precis. Future Med. 1, 10–31. doi: 10.23838/pfm.2017.00101

[ref46] Leibig C. Allken V. Berens P. Wahl S. Strodthoff N. (2017). Leveraging uncertainty information from deep neural networks for disease detection. Sci. Rep. 7:17876. doi: 10.1038/s41598-017-17876-z PMC5736701 29259224

[ref47] Liu X. Rivera S. C. Moher D. Calvert M. J. Denniston A. K. Ashrafian H. . (2020). Reporting guidelines for clinical trial reports for interventions involving artificial intelligence: the CONSORT-AI extension. Lancet Digit. Health 2, e537–e548. doi: 10.1016/S2589-7500(20)30218-1, 33328048 PMC8183333

[ref48] Mahmood F. Hussain L. Al-Jumeily D. Du K. L. (Eds.) (2022). Artificial Intelligence in cancer Diagnosis and Prognosis: Volume 2: Breast and Bladder Cancer. Bristol, UK: IOP Publishing.

[ref49] Mayo-Wilson E. Li T. Fusco N. Dickersin K. MUDS investigators (2018). Practical guidance for using multiple data sources in systematic reviews and meta-analyses (with examples from the MUDS study). Res. Synth. Methods 9, 2–12. doi: 10.1002/jrsm.1277, 29057573 PMC5888128

[ref50] McDermid J. Pumfrey D. Habli I. (2021). Artificial intelligence explainability: the technical and ethical dimensions. Philos. Trans. R. Soc. A Math. Phys. Eng. Sci. 379:20200363. doi: 10.1098/rsta.2020.0363 PMC8366909 34398656

[ref51] Meenakshi S. K. Saraswathi V. Malathy M. Subbulakshmi G. (2025). “Explainable quantum artificial intelligence for enhanced radiology decision support,” in 2025 IEEE International Conference on Emerging Trends in Engineering and Computing (ETECOM), (Riffa, Bahrain: IEEE), 1–7.

[ref52] Michelucci U. (2024). Fundamental Mathematical Concepts for Machine Learning in Science. Cham, Switzerland: Springer, 1–242. doi: 10.1007/978-3-031-56431-4

[ref53] Morfaki C. (2026). From algorithmic performance to hospital value: a narrative review of clinical artificial intelligence. Hospitals 3:19. doi: 10.3390/hospitals3040019

[ref54] Murphy K. P. (2022). Probabilistic Machine Learning: An Introduction. Cambridge, MA: MIT Press. Available online at: https://probml.github.io/book1 (accessed May 9, 2026).

[ref55] Oliveira T. A. Teodoro M. F. (2024). “Mathematical and statistical frameworks fostering advances in AI systems and computing,” in Computational Science and Its Applications – ICCSA 2024 Workshops, eds. Gervasi O. Murgante B. Garau C. Taniar D. Rocha A. M. A. Lago M. N., vol. 14816 (Cham: Springer).

[ref56] Page M. J. McKenzie J. E. Bossuyt P. M. Boutron I. Hoffmann T. C. Mulrow C. D. . (2021). The PRISMA 2020 statement: an updated guideline for reporting systematic reviews. BMJ 372:n71. doi: 10.1136/bmj.n71 PMC8005924 33782057

[ref4] Pantsar M. (2024). Theorem proving in artificial neural networks: new frontiers in mathematical AI. Euro. Jnl. Phil. Sci. 14: 4. doi: 10.1007/s13194-024-00569-6

[ref57] Parekh V. S. Jacobs M. A. (2019). Deep learning and radiomics in precision medicine. Expert Rev. Precis. Med. Drug Dev. 4, 59–72. doi: 10.1080/23808993.2019.1585805, 31080889 PMC6508888

[ref58] Pira L. Ferrie C. (2024). On the interpretability of quantum neural networks. Quantum Mach. Intell. 6:52. doi: 10.1007/s42484-024-00191-y

[ref59] Rani M. K. (2024). Linear algebra as the mathematical foundation of artificial intelligence: concepts, applications, and future prospects. Int. J. Eng. Sci. Humanit. 14, 123–137. doi: 10.62904/bmwhds52

[ref60] Ray S. Bhattacharya P. Mattar E. A. Mukhopadhyay A. (2025). Coalition of explainable artificial intelligence and quantum computing in precision medicine. Comput. Struct. Biotechnol. J. 27, 5234–5251. doi: 10.1016/j.csbj.2025.11.031, 41340890 PMC12671375

[ref61] Reyna M. A. Nsoesie E. O. Clifford G. D. (2022). Rethinking algorithm performance metrics for artificial intelligence in diagnostic medicine. JAMA 328, 329–330. doi: 10.1001/jama.2022.10561, 35802382 PMC11798426

[ref62] Rudin C. (2019). Stop explaining black box machine learning models for high stakes decisions and use interpretable models instead. Nat. Mach. Intell. 1, 206–215. doi: 10.1038/s42256-019-0048-x, 35603010 PMC9122117

[ref63] Russell S. Norvig P. (2021). Artificial Intelligence: A modern Approach. 4th Edn Hoboken, NJ: Pearson.

[ref64] Salvi D. Mazzetti M. Campagner A. Cabitza F. (2025). Explainability and uncertainty: two sides of the same coin for enhancing the interpretability of deep learning models in healthcare. Int. J. Med. Inform. 196:105846. doi: 10.1016/j.ijmedinf.2025.105846 39993336

[ref65] Schott B. Pinchuk D. Santoro-Fernandes V. Klaneček Ž. Rivetti L. Deatsch A. . (2024). Uncertainty quantification via localized gradients for deep learning-based medical image assessments. Phys. Med. Biol. 69:155015. doi: 10.1088/1361-6560/ad611d, 38981594 PMC13037787

[ref66] Shakeri E. de Souza Santos R. Far B. (2026). Fairness definitions and metrics in deep reinforcement learning for drug discovery in healthcare: a rapid evidence review. In: 2026 IEEE 50th Annual Computers, Software, and Applications Conference (COMPSAC). Piscataway, NJ: IEEE. 1112–1121.

[ref67] Shakeri E. Far B. (2026). Artificial intelligence in healthcare and drug discovery: a scoping review of current trends, challenges, and future directions. Intell. Based Med. 15:100442. doi: 10.1016/j.ibmed.2026.100442, 42574925

[ref68] Shamshad F. Khan S. Zamir S. W. Khan M. H. Hayat M. Khan F. S. . (2023). Transformers in medical imaging: a survey. Med. Image Anal. 88:102802. doi: 10.1016/j.media.2023.102802, 37315483

[ref69] Shea B. J. Reeves B. C. Wells G. Thuku M. Hamel C. Moran J. . (2017). AMSTAR 2: a critical appraisal tool for systematic reviews that include randomized or non-randomized studies of healthcare interventions, or both. BMJ 358:j4008. doi: 10.1136/bmj.j4008 PMC5833365 28935701

[ref70] Shoemaker K. Ger R. Court L. E. Aerts H. Vannucci M. Peterson C. B. (2023). Bayesian feature selection for radiomics using reliability metrics. Front. Genet. 14:1112914. doi: 10.3389/fgene.2023.1112914 PMC10030957 36968604

[ref71] Simen A. Flores-Garrigos C. Hegade N. N. Montalban I. Vives-Gilabert Y. Michon E. . (2024). Digital-analog quantum convolutional neural networks for image classification. Phys. Rev. Res. 6:L042060. doi: 10.1103/PhysRevResearch.6.L042060

[ref72] Stone J. V. (2024). Artificial Intelligence Engines: A Tutorial Introduction to the Mathematics of deep Learning. United States: Packt Publishing Ltd.

[ref73] Stovold E. Beecher D. Foxlee R. Noel-Storr A. (2014). Study flow diagrams in Cochrane systematic review updates: an adapted PRISMA flow diagram. Syst. Rev. 3:54. doi: 10.1186/2046-4053-3-54, 24886533 PMC4046496

[ref74] Suh N. Cheng G. (2024). A survey on statistical theory of deep learning: approximation, training dynamics, and generative models. arXiv, arXiv:2401.07187. doi: 10.48550/arXiv.2401.07187

[ref75] Symeonidis P. Zioupos A. (2016). “Performing SVD on matrices and its extensions,” in Matrix and Tensor Factorization Techniques for Recommender Systems, (Cham: Springer).

[ref76] Van Eck N. J. Waltman L. (2010). Software survey: VOSviewer, a computer program for bibliometric mapping. Scientometrics 84, 523–538. doi: 10.1007/s11192-009-0146-3, 20585380 PMC2883932

[ref77] van Timmeren J. Cester D. Tanadini-Lang S. Alkadhi H. Baessler B. (2020). Radiomics in medical imaging—“how-to” guide and critical reflection. Insights Imaging 11:91. doi: 10.1186/s13244-020-00887-2 PMC7423816 32785796

[ref78] Vasey B. Nagendran M. Campbell B. Clifton D. A. Collins G. S. Denaxas S. . (2022). Reporting guideline for the early-stage clinical evaluation of decision support systems driven by artificial intelligence: DECIDE-AI. Nat. Med. 28, 924–933. doi: 10.1038/s41591-022-01772-9, 35585198

[ref79] Wachter S. Mittelstadt B. Russell C. (2017). Counterfactual explanations without opening the black box: automated decisions and the GDPR. Harv. J. Law Technol. 31, 841–887. doi: 10.2139/ssrn.3063289

[ref80] Wei L. Liu H. Xu J. Shi L. Shan Z. Zhao B. . (2023). Quantum machine learning in medical image analysis: a survey. Neurocomputing 525, 42–53. doi: 10.1016/j.neucom.2023.01.049

[ref81] Weihs C. Ickstadt K. (2018). Data science: the impact of statistics. Int. J. Data Sci. Anal. 6, 189–194. doi: 10.1007/s41060-018-0102-5

[ref82] Widdows D. Kitto K. Cohen T. (2021). Quantum mathematics in artificial intelligence. J. Artif. Intell. Res. 72, 1307–1341. doi: 10.1613/jair.1.12702

[ref83] World Health Organization (2024). Ethics and Governance of Artificial Intelligence for Health: Large Multi-Modal Models. WHO Guidance. Geneva, Switzerland: World Health Organization.

[ref84] Wright S. J. Recht B. (2022). Optimization for Data Analysis. Cambridge, UK: Cambridge University Press.

[ref85] Wu Z. Pan S. Chen F. Long G. Zhang C. Yu P. S. (2021). A comprehensive survey on graph neural networks. IEEE Trans. Neural Networks Learn. Syst. 32, 4–24. doi: 10.1109/TNNLS.2020.2978386, 32217482

[ref86] Yan F. Huang H. Pedrycz W. Hirota K. (2024). Review of medical image processing using quantum-enabled algorithms. Artif. Intell. Rev. 57:300. doi: 10.1007/s10462-024-10932-x

[ref87] Yepes-Nuñez J. J. Urrútia G. Romero-García M. Alonso-Fernández S. (2021). The PRISMA 2020 statement: an updated guideline for reporting systematic reviews Declaración PRISMA 2020: una guía actualizada Para la publicación de revisiones sistemáticas. Rev. Esp. Cardiol. 74, 790–799. doi: 10.1016/j.recesp.2021.06.016, 34446261

[ref88] Żądło T. Chwila A. (2025). A step towards the integration of machine learning and classic model-based survey methods. Int. J. Mach. Learn. Cybern. 16, 8177–8194. doi: 10.1007/s13042-025-02718-6

[ref89] Zhang Y. (2022). An introduction to matrix factorization and factorization machines in recommendation system, and beyond. arXiv. arXiv:2203.11026. doi: 10.48550/arXiv.2203.11026

[ref90] Zhang S. Tong H. Xu J. Maciejewski R. (2019). Graph convolutional networks: a comprehensive review. Comput. Soc. Netw. 6, 1–23. doi: 10.1186/s40649-019-0069-y, 37915858 PMC10615927

[ref91] Zhovannik I. Bontempi D. Romita A. Pfaehler E. Primakov S. Dekker A. . (2022). Segmentation uncertainty estimation as a sanity check for image biomarker studies. Cancer 14:1288. doi: 10.3390/cancers14051288, 35267597 PMC8909427

[ref92] Zwanenburg A. Vallières M. Abdalah M. A. Aerts H. J. W. L. Andrearczyk V. Apte A. . (2020). The image biomarker standardization initiative: standardized quantitative Radiomics for high-throughput image-based phenotyping. Radiology 295, 328–338. doi: 10.1148/radiol.2020191145, 32154773 PMC7193906

