# Supplementary material for: Mathematical foundations of artificial intelligence in healthcare: a systematic review
Source: Front Artif Intell. 2026 Oct 21;9:1995929. doi: 10.3389/frai.2026.1995929 (PMC13731370; doi:10.3389/frai.2026.1995929)
Supplement: Supplementary file 1 [file Data_Sheet_1.zip › Appendix/APPENDIX F PROSPERO.pdf]

NIHR | National Institute for Health and Care Research

PROSPERO
International prospective register of systematic reviews

Mathematical Foundations of Artificial Intelligence for Healthcare: A Systematic Review

Yuliana Jiménez-Gaona, Vasudevan Lakshminarayanan

REVIEW TITLE AND BASIC DETAILS

Review title
Mathematical Foundations of Artificial Intelligence for Healthcare: A Systematic Review

Review type
None

Condition or domain being studied
Artificial intelligence; Machine learning; Healthcare quality assessment

Rationale for the review
Artificial intelligence is increasingly applied across healthcare, including medical imaging, radiomics, diagnosis, risk prediction, and clinical decision support. However, existing reviews generally focus on specific AI techniques, clinical applications, explainable AI, or emerging areas such as quantum machine learning, while the mathematical foundations underlying these approaches are often examined separately. Consequently, there is limited integrated evidence explaining how classical and emerging mathematical domains collectively support the development, validation, interpretability, robustness, and clinical reliability of healthcare AI systems.

This systematic review addresses this gap by synthesizing evidence on mathematical foundations including linear algebra, calculus, optimization, probability and statistics, information theory, graph theory, stochastic processes, random matrix theory, and quantum computing, and examining their roles in contemporary healthcare AI. The review will provide a unified framework linking mathematical theory with AI methodologies and healthcare applications, while identifying methodological limitations, underexplored mathematical domains, and future directions for developing robust, explainable, uncertainty-aware, and clinically trustworthy AI systems.

Review objectives
• ¿Which mathematical domains most strongly support AI healthcare applications?

• ¿How are emerging mathematical frameworks changing explainability and uncertainty estimation?

• ¿How are mathematical foundations associated with different AI architectures and healthcare applications?

• ¿What methodological gaps currently limit mathematically rigorous, trustworthy, and clinically translatable healthcare AI?

Keywords
Artificial Intelligence; Mathematical Foundations; Healthcare

Country
Sweden

ELIGIBILITY CRITERIA

Population

Included
• Original research articles, review articles, and systematic reviews published in peer-reviewed journals about mathematical foundations supporting AI algorithms, including but not limited to linear algebra, calculus, optimization, probability theory, statistics, information theory, graph theory, stochastic processes, random matrix theory, and quantum computing.

• Studies reporting the use of mathematical models, simulations, algorithms, or computational frameworks in healthcare, medical imaging, radiomics, biomedical signal processing, clinical decision support systems, or precision medicine.

• Studies evaluating AI model performance, interpretability, explainability, uncertainty quantification, robustness, fairness, transparency, or ethical AI frameworks.

• Articles written in English between January 2016 and December 2026.

Excluded
• Conference abstracts, editorials, letters to the editor, commentaries, opinion papers, book reviews, dissertations, patents, and non-peer-reviewed publications.

• Studies unrelated to mathematical content, Artificial Intelligence, machine learning, deep learning, mathematical modeling, or healthcare applications.

• Articles with insufficient methodological information, incomplete data, or lacking a clear methodological or mathematical framework.

• Publications focused exclusively on software implementation or engineering aspects without discussing the underlying mathematical principles.

• Duplicate records identified across databases.

• Studies published before 2016.

• Articles published in languages other than English.

Intervention(s) or exposure(s)

Included
Health Assessment; Artificial intelligence; Medical Image Management System Application Software; Clinical Decision Rules; Clinical Support

Comparator(s) or control(s)

This review does not have any comparators

Study design

Only randomized study types will be included.

Included

This review will include original research articles, review articles, and systematic reviews published in peer-reviewed journals. Eligible studies will investigate mathematical foundations supporting artificial intelligence algorithms, including linear algebra, calculus, optimization, probability and statistics, information theory, graph theory, stochastic processes, random matrix theory, and quantum computing. Studies applying mathematical models, simulations, algorithms, or computational frameworks to healthcare domains, including medical imaging, radiomics, biomedical signal processing, clinical decision support, and precision medicine, will also be included. Only studies published in English between 2016 and 2026 will be considered.

Context

This review is conducted in the context of artificial intelligence applications in healthcare, with a specific focus on the mathematical foundations that support the development, optimization, evaluation, interpretability, and clinical translation of AI models. The review covers healthcare domains including medical imaging, radiomics, biomedical signal processing, clinical decision support, diagnosis, and precision medicine. It examines classical and emerging mathematical frameworks, including linear algebra, calculus, optimization, probability and statistics, information theory, graph theory, stochastic processes, random matrix theory, and quantum computing, and their contribution to robust, explainable, and clinically trustworthy AI systems.

TIMELINE OF THE REVIEW

Date of first submission to PROSPERO

30 September 2026

Review timeline

Review start date: 24 August 2026. Review end date: 23 December 2026.

Date of registration in PROSPERO

01 October 2026

AVAILABILITY OF FULL PROTOCOL

Availability of full protocol

A full protocol has been written and uploaded to PROSPERO. The protocol may be accessed through this link https://www.crd.york.ac.uk/PROSPEROFILES/0c14472f072c32a77f5018da08c84b66.pdf.

SEARCHING AND SCREENING

Search for unpublished studies

Only published studies will be sought.

Main sources that will be searched

The main sources to be searched are *Google Scholar*, *MEDLINE*, *PubMed* and *Scopus*.

Search language restrictions

The review will only include studies published in Spanish.

Search date restrictions

Databases will be searched for articles published from 4 January 2016 and before by 4 December 2026.

Other methods of identifying studies

Other studies will be identified by: *contacting authors or experts*, *looking through all the articles that cite the papers included in the review ("snowballing" or forward citation searching)*, *reference list checking (backward citation searching)* and *searching conference proceedings*.

Link to search strategy

A full search strategy has been uploaded to PROSPERO. The PDF may be accessed through this link
https://www.crd.york.ac.uk/PROSPEROFILES/6b581be8b815b5b8363a8dc828d9971f.pdf.

Selection process

Studies will be screened independently by at least two people (or person/machine combination) with a process to resolve differences.

Other relevant information about searching and screening

Two reviewers independently screened titles and abstracts, followed by full-text eligibility assessment. Disagreements were resolved through discussion and consensus between the reviewers.

DATA COLLECTION PROCESS

Data extraction from published articles and reports

Data will be extracted independently by at least two people (or person/machine combination) with a process to resolve differences.

Authors will be asked to provide any required data not available in published reports.

Study risk of bias or quality assessment

Risk of bias will be assessed using: *AMSTAR-2* and *JBI tools*

Data will be assessed independently by at least two people (or person/machine combination) with a process to resolve differences.

Additional information will be sought from study investigators if required information is unclear or unavailable in the study publications/reports.

Reporting bias assessment

JBI, AMSTAR 2

Certainty assessment

Confidence in the body of evidence to be assessed based on the methodological quality, consistency, and robustness of the included studies. Methodological quality is independently

evaluated by two reviewers using the Joanna Briggs Institute (JBI) Critical Appraisal Tools adapted to the corresponding study designs. Systematic reviews is additionally be assessed using AMSTAR-2. Studies will be classified as high quality (≥80%), moderate quality (60–79%), or low quality (<60%) according to the predefined appraisal criteria. Confidence in the synthesized evidence is also consider consistency of findings across studies, methodological rigor, adequacy of AI model validation, transparency of mathematical and statistical methods, and limitations affecting generalizability and reproducibility. Disagreements between reviewers to be resolved through discussion and consensus. No formal GRADE assessment is planned because of the substantial methodological and clinical heterogeneity of the included evidence and the non-interventional nature of the review.

OUTCOMES TO BE ANALYSED

Main outcomes

The main outcomes are the mathematical domains underpinning healthcare AI and their roles in model development, optimization, uncertainty estimation, interpretability, robustness, validation, and clinical application. Secondary outcomes include the distribution of AI architectures, healthcare application areas, clinical tasks, and performance metrics reported across the included studies.

Additional outcomes

There are no additional outcomes.

PLANNED DATA SYNTHESIS

Strategy for data synthesis

A formal narrative and descriptive synthesis will be conducted in accordance with PRISMA 2020 reporting principles. Meta-analysis is not planned because substantial methodological and clinical heterogeneity is expected across the included studies in terms of mathematical foundations, artificial intelligence architectures, healthcare applications, datasets, validation strategies, and reported performance measures.

Included studies will first be systematically characterized according to publication year, study design, mathematical domain, AI methodology, healthcare application, dataset characteristics, validation approach, evaluation metrics, and methodological quality. Evidence will then be grouped into major mathematical domains, including linear algebra, calculus and optimization, probability and statistics, information theory, graph theory and discrete mathematics, numerical analysis, stochastic processes, random matrix theory, and emerging mathematical paradigms such as quantum computing.

Within each domain, findings will be synthesized using an integrative framework linking: mathematical foundation → mathematical concept or method → function within the AI model → AI architecture → healthcare application → evaluation and validation → implications for robustness, uncertainty, interpretability, and clinical translation.

Quantitative descriptive synthesis will be used where appropriate to summarize frequencies and proportions of mathematical domains, AI architectures, healthcare applications, clinical tasks, and commonly reported evaluation metrics. Performance measures such as accuracy, sensitivity, specificity, precision, recall, F1-score, AUC/ROC, Dice coefficient, IoU, MSE, PSNR, SSIM, and other task-specific measures will be extracted when reported. However, these estimates will not be statistically pooled unless sufficiently homogeneous groups of studies are identified.

A complementary bibliometric synthesis will be performed using VOSviewer (version 1.6.20) to examine publication trends, author collaboration, keyword co-occurrence, citation patterns, and thematic research clusters. Association-strength normalization will be used for network visualization. Bibliometric findings will be interpreted alongside the systematic evidence synthesis to identify established and emerging mathematical themes in healthcare AI.

Methodological quality will inform interpretation of the synthesized evidence. Studies will be appraised using the Joanna Briggs Institute (JBI) Critical Appraisal Tools, with systematic reviews additionally assessed using AMSTAR-2. Where appropriate, patterns will be compared across methodological-quality categories to determine whether the principal conclusions remain consistent among higher-quality studies.

Heterogeneity will primarily be assessed qualitatively by comparing mathematical methods, AI architectures, healthcare domains, datasets, validation procedures, and outcome definitions. Statistical heterogeneity measures such as I^2 will only be considered if a sufficiently homogeneous subset of studies permits quantitative pooling. If such homogeneity is not identified, no meta-analysis will be undertaken.

The final synthesis will identify areas of convergence and inconsistency across the literature, underrepresented mathematical foundations, methodological limitations, and research gaps relevant to the development of robust, explainable, uncertainty-aware, reproducible, and clinically trustworthy artificial intelligence systems in healthcare.

CURRENT REVIEW STAGE

Stage of the review at this submission

Review stage	Started	Completed
Pilot work	✓	✓
Formal searching/study identification	✓	✓
Screening search results against inclusion criteria	✓	✓
Data extraction or receipt of IPD		
Risk of bias/quality assessment		
Data synthesis		

Review status
The review is currently planned or ongoing.

Publication of review results
Results of the review will be published in English.

Journal publication
Not yet published in a journal but will be in future.

REVIEW AFFILIATION, FUNDING AND PEER REVIEW

Review team members
Associate Professor Yuliana Jiménez-Gaona (review guarantor and contact) ORCID: 0000-0001-7155-5546. Universidad Técnica Particular de Loja. Ecuador.

No conflict of interest declared.

Professor Vasudevan Lakshminarayanan. University of Waterloo. Canada.

No conflict of interest declared.

Named contact
Associate Professor Yuliana Jiménez-Gaona (ydjimenez@utpl.edu.ec). ORCID: 0000-0001-7155-5546. Universidad Técnica Particular de Loja. Ecuador.

Review affiliation
1Departamento de Química y Producción, Universidad Técnica Particular de Loja, San Cayetano Alto s/n CP1101608, Loja, Ecuador, ydjimenez@utpl.edu.ec

Funding source

Additional non-commercial funding information
UTPL

Peer review
There has been no peer review of this planned review.

ADDITIONAL INFORMATION

Review conflict of interest
Declared individual interests are recorded under team member details.. No additional interests are recorded for this review.

Medical Subject Headings
Artificial Intelligence; Classification; Delivery of Health Care; Humans

SIMILAR REVIEWS

Check for similar records already in PROSPERO
Although existing PROSPERO records address artificial intelligence and machine learning in healthcare, they primarily focus on specific clinical applications, diagnostic performance, implementation, education, or particular medical specialties. The present review has a distinct

scope and research question, as it systematically investigates the classical and emerging mathematical foundations underlying healthcare AI, including linear algebra, calculus, optimization, probability and statistics, information theory, graph theory, stochastic processes, random matrix theory, and quantum computing. It further examines how these mathematical domains support model development, optimization, evaluation, interpretability, robustness, uncertainty quantification, and clinical translation. Therefore, the proposed review complements rather than duplicates existing registered reviews.

PROSPERO version history

- Version 1.0, published 01 Oct 2026

Disclaimer

The content of this record displays the information provided by the review team. PROSPERO does not peer review registration records or endorse their content.

PROSPERO accepts and posts the information provided in good faith; responsibility for record content rests with the review team. The guarantor for this record has affirmed that the information provided is truthful and that they understand that deliberate provision of inaccurate information may be construed as scientific misconduct.

PROSPERO does not accept any liability for the content provided in this record or for its use. Readers use the information provided in this record at their own risk.

Any enquiries about the record should be referred to the named review contact
