# Supplementary material for: Mathematical foundations of artificial intelligence in healthcare: a systematic review
Source: Front Artif Intell. 2026 Oct 21;9:1995929. doi: 10.3389/frai.2026.1995929 (PMC13731370; doi:10.3389/frai.2026.1995929)
Supplement: Supplementary file 1 [file Data_Sheet_1.zip › Appendix/APPENDIX A.docx]

**APPENDIX A**

**Table 1. Exact search strings used in Publish or Perish with Google Scholar as the data source**

*Search period: 2016–2026. Data source: Google Scholar via Harzing’s Publish or Perish. Export format: RIS.*

| **Search** | **Mathematical foundation term** | **AI terms** | **Healthcare terms** |
| --- | --- | --- | --- |
| S1 | "mathematical foundation" | "artificial intelligence" OR "machine learning" OR "deep learning" | "healthcare" OR "medicine" OR "biomedical" OR "radiology" OR "radiomics" |
| S2 | "linear algebra" |  |  |
| S3 | "calculus" OR "optimization" |  |  |
| S4 | "probability" OR "statistics" |  |  |
| S5 | "information theory" OR "graph theory" |  |  |
| S6 | "discrete mathematics" OR "numerical analysis" |  |  |
| S7 | "stochastic mathematics" |  |  |
| S8 | "quantum mathematics" OR "quantum computing" |  |  |

*Note: These strings should be reported as “exact search strings” only if they correspond to the queries actually executed in Publish or Perish. If different fields, limits, or combinations were used, the table should be updated to match the original search log.*

**Table 2. Scopus Global semantic structure search using Boolean operators and key words to determine the main research’s focusing on the main objectives.**

| **Topic** | **Category** | **Boolean structure** | **Keywords Search** |
| --- | --- | --- | --- |
| Mathematical foundations of AI, analyzing how core mathematical disciplines, and emerging fields support modeling, simulation, and medicine applications. | Mathematical foundations of AI.  Mathematical disciplines, Modeling, Simulation, Medicine applications. | AND, OR " | “Artificial Intelligence” OR “AI” AND “AI terms” AND “Health terms” AND Imaging” AND Diagnostics” AND Deases” AND “Cancers” AND  AND “Medical Image Analysis” AND “Biomedical Signal”  AND “Image Processing” AND “Signal Processing” AND “Biological Engineering & Computing”  AND “Math applications in healthcare” AND “Mathematical foundations” OR “AI health Applications” AND “modeling” OR “simulation”. |
| The role of fundamental mathematical domains, including linear algebra, calculus, optimization, probability, statistics, and discrete mathematics in the development, functioning, and performance of AI algorithms. | Linear Algebra     Calculus    Probability  and Statistics    Discrete mathematics and information theory    AI mathematic modeling, simulation.    AI in medicine applications. | AND, OR | “Math domains” AND “Linear algebra” OR “Calculus” OR “Optimization” OR “Probability” OR “Statistics ” OR “discrete mathematics” AND “Deep Learning” AND “Machine Learning” AND “AI algorithms ” OR “AI architectures” AND “DL Models” AND  “ML models” AND “AI Health Application” AND “Mathematical principles”  OR “Mathematical theory” AND “computer vision” AND  “AI health Applications” |
| Mathematical principles contribute to major AI applications such as computer vision, natural language processing, reinforcement learning, and advanced architectures including CNNs, GANs, and Transformers. | Mathematical Foundations  Healthcare Application  Computer vision,  Algorithms,  Architectures,  Future Mathematical Challenges | AND, OR | "mathematical principles" OR "mathematical foundations" OR "mathematical modeling" OR "applied mathematics"OR "machine learning" OR "deep learning" OR” "CNN" OR "convolutional neural network*" OR "GAN" OR "generative adversarial network*" OR "Transformer*"computer vision" OR "natural language processing" OR "reinforcement learning" "healthcare" OR "medical imaging" OR "clinical decision support" OR "radiomics". |
| The main contribution of mathematical modeling and simulation in enhancing interpretability, transparency, fairness, and ethical alignment. | Mathematical modeling,  Mathematical simulation , Emerging mathematics,  Ethical design  Responsible IA,  Healthcare AI, Clinical decision support, Radiomics. | AND | "mathematical modeling" OR ¨simulation¨ AND "AI systems" AND ¨interpretability¨ OR ¨explainability¨ OR ¨transparency¨ OR ¨ethics¨ OR "responsible AI" AND "healthcare AI" OR ¨radiomics¨ AND ¨ ¨robustness¨ AND "mathematical challenges" |
