# Supplementary material for: Mathematical foundations of artificial intelligence in healthcare: a systematic review
Source: Front Artif Intell. 2026 Oct 21;9:1995929. doi: 10.3389/frai.2026.1995929 (PMC13731370; doi:10.3389/frai.2026.1995929)
Supplement: Supplementary file 1 [file Data_Sheet_1.zip › Appendix/APPENDIX B.docx]

**APPENDIX B. Reviewer Rubric for Study Screening and Methodological Quality Assessment**

*PRISMA 2020 selection workflow and JBI-based appraisal*

| **Reviewer name** |  | **Reviewer ID** |  |
| --- | --- | --- | --- |
| **Study citation / ID** |  | **Review date** |  |
| **Review stage** | ☐ Title/abstract  ☐ Full text  ☐ Quality appraisal | **Version** |  |

**Instructions.** Complete the rubric independently. Apply the predefined eligibility criteria consistently, record a justification for exclusions, and avoid resolving disagreements until both reviewers have completed their initial assessment. Disagreements should be resolved by discussion and, if required, by a third reviewer.

# 1. Eligibility Screening Rubric

| **No.** | **Criterion** | **Operational decision rule** | **Yes** | **No** | **Unclear / notes** |
| --- | --- | --- | --- | --- | --- |
| 1 | Relevant topic | Addresses mathematical foundations, mathematical models, simulation, machine learning, deep learning, or AI methodology relevant to healthcare or biomedical applications. | ☐ | ☐ |  |
| 2 | Healthcare relevance | Includes healthcare, medical imaging, radiomics, biomedical signal processing, clinical decision support, precision medicine, diagnosis, prognosis, or a clearly transferable healthcare application. | ☐ | ☐ |  |
| 3 | Eligible publication type | Peer-reviewed original research, review article, or systematic review. Excludes editorials, letters, abstracts, commentaries, opinion papers, dissertations, patents, and book reviews. | ☐ | ☐ |  |
| 4 | Publication period | Published within the predefined review period. | ☐ | ☐ |  |
| 5 | Language | Published in English. | ☐ | ☐ |  |
| 6 | Methodological sufficiency | Provides sufficient methodological, mathematical, analytical, or computational information for appraisal. | ☐ | ☐ |  |
| 7 | Not software-only | Does not focus exclusively on software or engineering implementation without discussion of underlying mathematical principles. | ☐ | ☐ |  |
| 8 | Duplicate status | Not a duplicate of another record already retained. | ☐ | ☐ |  |

**Screening decision:** ☐ Include	☐ Exclude	☐ Seek clarification / full text

**Primary exclusion reason (select one):** ☐ Wrong topic	☐ No healthcare relevance	☐ Ineligible publication type	☐ Outside date range	☐ Non-English	☐ Insufficient methods	☐ Duplicate	☐ Other

**Reviewer Sign:** ________________________________________
