# Supplementary material for: Mathematical foundations of artificial intelligence in healthcare: a systematic review
Source: Front Artif Intell. 2026 Oct 21;9:1995929. doi: 10.3389/frai.2026.1995929 (PMC13731370; doi:10.3389/frai.2026.1995929)
Supplement: Supplementary file 1 [file Data_Sheet_1.zip › Appendix/Appendix C.docx]

**APPENDIX C. Methodological Quality Assessment**

**Table 3. Representative methodological quality assessment of studies included in the systematic review**

| **Code** | **Study** | **Study type / principal focus** | **Applicable criteria (k)** | **Maximum score (2k)** | **Score obtained** | **Quality score (%)** | **Quality category** |
| --- | --- | --- | --- | --- | --- | --- | --- |
| S065 | Parekh & Jacobs (2019) | Review - deep learning and radiomics in precision medicine | 10 | 20 | 13 | 65.0 | Moderate |
| S066 | Barredo Arrieta et al. (2020) | Review - explainable and responsible AI | 10 | 20 | 13 | 65.0 | Moderate |
| S046 | van Timmeren et al. (2020) | Review/guidance - radiomics methodology and reproducibility | 10 | 20 | 16 | 80.0 | High |
| S082 | Zwanenburg et al. (2020) | Methodological/standardization study - quantitative radiomics | 10 | 20 | 16 | 80.0 | High |
| S051 | Faghani et al. (2023) | Review/methodological - uncertainty quantification in radiological AI | 10 | 20 | 15 | 75.0 | Moderate |
| S054 | Shamshad et al. (2023) | Survey/review - Transformers in medical imaging | 10 | 20 | 15 | 75.0 | Moderate |
| S093 | Chakraborti et al. (2025) | Methodological - personalized uncertainty quantification | 10 | 20 | 13 | 65.0 | Moderate |
| S055 | Huang et al. (2025) | Empirical clinical AI - radiomics and quantum machine learning | 12 | 24 | 18 | 75.0 | Moderate |
| S092 | Salvi et al. (2025) | Review/methodological - explainability and uncertainty in healthcare AI | 10 | 20 | 15 | 75.0 | Moderate |

**Scoring rule.** Yes = 2; Partially/Unclear = 1; No = 0; N/A = excluded from the denominator.

**Normalized quality score:** Quality score (%) = Score obtained / (2 x k) x 100, where k is the number of applicable appraisal criteria.

**Quality classification.** High >=80%; Moderate = 60-79%; Low <60%.

**Note.** Scores were derived using the adapted 12-domain JBI-based appraisal described in Appendix D. Criteria not applicable to a particular study design were excluded from the denominator. Criteria for which sufficient information could not be established from the available abstract/bibliographic information were scored as Partially/Unclear rather than assumed to be satisfied. These representative scores should be verified against the full text before final submission.
