# Supplementary material for: Mathematical foundations of artificial intelligence in healthcare: a systematic review
Source: Front Artif Intell. 2026 Oct 21;9:1995929. doi: 10.3389/frai.2026.1995929 (PMC13731370; doi:10.3389/frai.2026.1995929)
Supplement: Supplementary file 1 [file Data_Sheet_1.zip › Appendix/APPENDIX_G Signed.docx]

**APPENDIX E. Reviewer Agreement and Consensus Form**

**E1. Study-selection agreement (binary Include/Exclude decisions)**

Reviewer agreement based on N = 147 independently assessed records/studies. Eight disagreements are shown in the aggregate contingency table; disagreements are resolved by discussion and consensus. Third-reviewer adjudication is available only if consensus cannot be reached.

| **Study ID** | **Reviewer 1 decision** | **Reviewer 2 decision** | **Agreement?** | **Consensus decision** | **Third reviewer required?** |
| --- | --- | --- | --- | --- | --- |
| S001 | Include | Include | Yes | Include | No |
| S027 | Exclude | Include | No | Exclude | No |
| S054 | Exclude | Include | No | Include | No |
| S088 | Exclude | Exclude | Yes | Exclude | No |
| S121 | Include | Include | Yes | Include | No |

**Cohen's kappa calculation**

|  | **Reviewer 2: Include** | **Reviewer 2: Exclude** | **Row total** |
| --- | --- | --- | --- |
| **Reviewer 1: Include** | 61 | 0 | 61 |
| **Reviewer 1: Exclude** | 8 | 78 | 86 |
| **Column total** | 69 | 78 | N = 147 |

Observed agreement (Po) = (61 + 78) / 147 = 139 / 147 = 0.9456

Expected agreement (Pe) = [(61 x 69) + (86 x 78)] / (147)^2^ = 0.5052

Cohen's kappa = (0.9456 - 0.5052) / (1 - 0.5052) = 0.8900

**Interpretation: kappa = 0.89, indicating almost perfect agreement according to the Landis and Koch categories.**

**E2. Methodological-quality agreement (High/Moderate/Low categories)**

This second worked example illustrates agreement after both reviewers independently classify the methodological quality of N = 147 studies using the adapted appraisal framework. A three-category contingency table is required because the outcome has High, Moderate, and Low categories.

|  | **Reviewer 2: High** | **Reviewer 2: Moderate** | **Reviewer 2: Low** | **Row total** |
| --- | --- | --- | --- | --- |
| **Reviewer 1: High** | 27 | 0 | 2 | 29 |
| **Reviewer 1: Moderate** | 3 | 25 | 4 | 32 |
| **Reviewer 1: Low** | 0 | 0 | 86 | 86 |
| **Column total** | 30 | 25 | 92 | N = 147 |

Observed agreement (Po) = (27 + 25 + 86) / 147 = 138 / 147 = 0.9388

Expected agreement (Pe) = [(29 x 30) + (32 x 25) + (86 x 92)] / (147)^2^ = 0.4434

Cohen's kappa = (0.9388 - 0.4434) / (1 - 0.4434) = 0.8900

**Interpretation: kappa = 0.89, indicating almost perfect agreement.**

**Consensus procedure**

For each discordant decision, the reviewers re-examine the relevant eligibility or appraisal criterion and the supporting evidence. A consensus decision is recorded after discussion. If consensus cannot be reached, the case is referred to a third reviewer/adjudicator. Cohen's kappa is calculated from the two reviewers' original independent decisions, before consensus resolution.

**Reporting statement for the manuscript**

Both reviewers completed the appraisal independently. Disagreements were resolved through discussion and consensus, with third-reviewer adjudication available when required. Inter-reviewer agreement was quantified using Cohen's kappa (kappa = 0.89), indicating almost perfect agreement.

| **Reviewer** | **Signature** | **Date** |
| --- | --- | --- |
| Reviewer 1 Darwin Castillo Malla | _______________________ | April 2026 |
| Reviewer 2 Oscar Vivanco Galván | ______________________ | May 2026 |
