# Supplementary material for: Mathematical foundations of artificial intelligence in healthcare: a systematic review
Source: Front Artif Intell. 2026 Oct 21;9:1995929. doi: 10.3389/frai.2026.1995929 (PMC13731370; doi:10.3389/frai.2026.1995929)
Supplement: Supplementary file 1 [file Data_Sheet_1.zip › Appendix/APPENDIX D.docx]

# APPENDIX D. JBI-Based Methodological Quality Appraisal

Scoring instructions. Assess each applicable criterion using a three-level scale: Yes = 2 points (criterion fully satisfied), Partially/Unclear = 1 point (criterion incompletely satisfied or reporting is insufficient for a definitive judgment), and No = 0 points (criterion not satisfied). Mark Not Applicable (N/A) when a criterion is not relevant to the study design; N/A criteria are excluded from the denominator. The normalized quality score is calculated as: Quality score (%) = [total points obtained / (2 × number of applicable criteria)] × 100. Studies are classified as high quality (≥80%), moderate quality (60–79%), or low quality (<60%).

| No. | Appraisal domain | Critical question | Yes (2) | No (0) | Partially / Unclear (1) | N/A | Comments / evidence |
| --- | --- | --- | --- | --- | --- | --- | --- |
| 1 | Study design and objectives | Are the research question, objectives, and study design clearly stated and appropriate? | ☐ | ☐ | ☐ | ☐ |  |
| 2 | Population or data source | Are the sample, dataset, data source, and selection procedures adequately described? | ☐ | ☐ | ☐ | ☐ |  |
| 3 | Mathematical framework | Are the mathematical concepts, assumptions, equations, or theoretical foundations clearly and correctly reported? | ☐ | ☐ | ☐ | ☐ |  |
| 4 | AI / computational methods | Are algorithms, model architecture, parameterization, preprocessing, and implementation procedures sufficiently described? | ☐ | ☐ | ☐ | ☐ |  |
| 5 | Data collection and measurement | Are data acquisition, variable definitions, labels, reference standards, and measurement procedures valid and reliable? | ☐ | ☐ | ☐ | ☐ |  |
| 6 | Bias and confounding | Are potential sources of selection bias, measurement bias, confounding, leakage, and class imbalance identified and addressed? | ☐ | ☐ | ☐ | ☐ |  |
| 7 | Statistical analysis | Are the statistical methods, uncertainty estimates, validation procedures, and performance metrics appropriate? | ☐ | ☐ | ☐ | ☐ |  |
| 8 | Model validation | Is internal, external, temporal, or cross-validation adequately reported and appropriate to the study aim? | ☐ | ☐ | ☐ | ☐ |  |
| 9 | Reproducibility and transparency | Are datasets, code, hyperparameters, model settings, or sufficient implementation details available for reproduction? | ☐ | ☐ | ☐ | ☐ |  |
| 10 | Results and reporting | Are results complete, internally consistent, clearly reported, and supported by tables, figures, or quantitative evidence? | ☐ | ☐ | ☐ | ☐ |  |
| 11 | Interpretation and limitations | Are conclusions supported by results, and are limitations, uncertainty, and generalizability appropriately discussed? | ☐ | ☐ | ☐ | ☐ |  |
| 12 | Clinical relevance and safety | Where applicable, are clinical utility, patient safety, fairness, subgroup performance, and deployment implications considered? | ☐ | ☐ | ☐ | ☐ |  |

**Study-type adaptation.** Apply all domains that are methodologically relevant to the study. For empirical AI/clinical studies, emphasize data source, measurement, bias/confounding, statistical analysis, model validation, performance evaluation, and clinical relevance. For methodological/computational studies, emphasize the mathematical framework, algorithmic specification, parameterization, reproducibility, and validation. For theoretical/foundational studies, patient-level data, clinical measurement, or clinical validation criteria may be marked N/A when genuinely inapplicable. Review articles should be judged on methodological transparency, synthesis procedures, reproducibility, and support for conclusions; systematic reviews are additionally assessed using AMSTAR-2. N/A must not be used for insufficient reporting; insufficiently reported applicable criteria should be rated Partially/Unclear or No, as appropriate.

**Scoring summary**

| **Score counts** | Yes (2): ____ Partially/Unclear (1): ____ No (0): ____ N/A: ____ |
| --- | --- |
| **Applicable criteria (k)** |  |
| **Total points obtained** |  |
| **Quality score (%)** | [Total points obtained / (2 x k)] x 100 = ______ % |
| **Quality category** | ☐ High (>=80%) ☐ Moderate (60-79%) ☐ Low (<60%) |
| **Reviewer confidence / concerns** | ☐ High ☐ Moderate ☐ Low Key methodological concerns: ______________________________________________ |
