# Supplementary material for: Mathematical foundations of artificial intelligence in healthcare: a systematic review
Source: Front Artif Intell. 2026 Oct 21;9:1995929. doi: 10.3389/frai.2026.1995929 (PMC13731370; doi:10.3389/frai.2026.1995929)
Supplement: Supplementary file 1 [file Data_Sheet_1.zip › Appendix/APPENDIX E.docx]

# APPENDIX E: Reviewer Agreement and Consensus Form

Use this form after both reviewers have independently completed screening and quality appraisal.

| **Study ID** | **Reviewer 1 decision** | **Reviewer 2 decision** | **Agreement?** | **Consensus decision** | **Third reviewer required?** |
| --- | --- | --- | --- | --- | --- |
|  |  |  | ☐ Yes ☐ No |  | ☐ Yes ☐ No |
|  |  |  | ☐ Yes ☐ No |  | ☐ Yes ☐ No |
|  |  |  | ☐ Yes ☐ No |  | ☐ Yes ☐ No |
|  |  |  | ☐ Yes ☐ No |  | ☐ Yes ☐ No |
|  |  |  | ☐ Yes ☐ No |  | ☐ Yes ☐ No |

**Cohen’s κ calculation**

Cohen’s kappa quantifies agreement beyond chance: κ = (Pₒ − Pₑ) / (1 − Pₑ), where Pₒ is observed agreement and Pₑ is expected agreement by chance.

|  | **Reviewer 2: Include** | **Reviewer 2: Exclude** | **Row total** |
| --- | --- | --- | --- |
| Reviewer 1: Include |  |  |  |
| Reviewer 1: Exclude |  |  |  |
| Column total |  |  | N = |

**Observed agreement (Pₒ):** __________________

**Expected agreement (Pₑ):** __________________

**Cohen’s κ:** __________________

| **κ range** | **Interpretation** | **Action** |
| --- | --- | --- |
| <0.00 | Poor | Reassess criteria and retrain reviewers |
| 0.00–0.20 | Slight | Clarify operational definitions |
| 0.21–0.40 | Fair | Conduct calibration exercise |
| 0.41–0.60 | Moderate | Review recurring disagreements |
| 0.61–0.80 | Substantial | Acceptable; document consensus process |
| 0.81–1.00 | Almost perfect | Strong reproducibility of decisions |

**Reported agreement for this review:** κ = 0.89, indicating almost perfect agreement according to the Landis and Koch interpretation.

| **Reviewer 1 signature** |  | **Date** |  |
| --- | --- | --- | --- |
| **Reviewer 2 signature** |  | **Date** |  |
| **Third reviewer / adjudicator** |  | **Date** |  |
